# Nitric oxide-driven modifications of lipoic arm inhibit α-ketoacid dehydrogenases

**DOI:** 10.1038/s41589-022-01153-w

**Published:** 2022-10-20

**Authors:** Gretchen L. Seim, Steven V. John, Nicholas L. Arp, Zixiang Fang, David J. Pagliarini, Jing Fan

**Affiliations:** 1 Morgridge Institute for Research, Madison, WI USA; 2 Department of Nutritional Sciences, University of Wisconsin-Madison, Madison, WI USA; 3 Cellular and Molecular Biology Graduate Program, University of Wisconsin-Madison, Madison, WI USA; 4 Department of Cell Biology and Physiology, Washington University School of Medicine, St. Louis, MO USA; 5 Department of Biochemistry and Molecular Biophysics, Washington University School of Medicine, St. Louis, MO USA; 6 Department of Genetics, Washington University School of Medicine, St. Louis, MO USA

**Keywords:** Metabolic pathways, Enzymes, Post-translational modifications, Immunology

## Abstract

Pyruvate dehydrogenase complex (PDHC) and oxoglutarate dehydrogenase complex (OGDC), which belong to the mitochondrial α-ketoacid dehydrogenase family, play crucial roles in cellular metabolism. These multi-subunit enzyme complexes use lipoic arms covalently attached to their E2 subunits to transfer an acyl group to coenzyme A (CoA). Here, we report a novel mechanism capable of substantially inhibiting PDHC and OGDC: reactive nitrogen species (RNS) can covalently modify the thiols on their lipoic arms, generating a series of adducts that block catalytic activity. *S*-Nitroso-CoA, a product between RNS and the E2 subunit’s natural substrate, CoA, can efficiently deliver these modifications onto the lipoic arm. We found RNS-mediated inhibition of PDHC and OGDC occurs during classical macrophage activation, driving significant rewiring of cellular metabolism over time. This work provides a new mechanistic link between RNS and mitochondrial metabolism with potential relevance for numerous physiological and pathological conditions in which RNS accumulate.

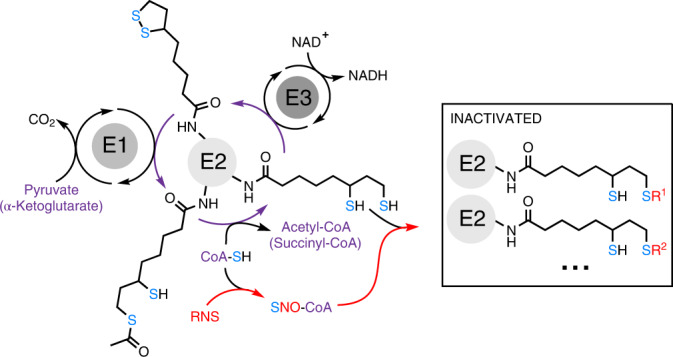

## Abstract (web summary)

Reactive nitrogen species can cause profound inhibition of α-ketoacid dehydrogenase complexes via covalent S-modifications of the E2 subunit’s catalytic lipoic arm. The enzymes’ substrate, CoA, can mediate targeted delivery of such modifications.

## Main

Mitochondrial α-ketoacid dehydrogenases are a family of multi-subunit enzyme complexes that includes PDHC, OGDC, and branched-chain ketoacid dehydrogenase complex (BCKDC). These enzymes function at critical crossroads in the metabolic network, controlling carbohydrate and amino acid metabolism, mitochondrial energy production and cellular redox state^1^. Specifically, PDHC catalyzes the conversion of pyruvate to acetyl-CoA, and OGDC catalyzes the oxidation of α-ketoglutarate (α-KG) to succinyl-CoA; thus, both control the entrance of major carbon sources into the tricarboxylic acid (TCA) cycle. α-Ketoacid dehydrogenase complexes share similar catalytic mechanisms involving coupled reactions with three subunits^1,2^. The E1 subunit decarboxylates an α-ketoacid and transfers the corresponding acyl group to a thiamine pyrophosphate cofactor. This acyl group is then transferred to the E2 subunit (dihydrolipoamide acyltransferase), which uses a lipoic arm covalently attached to a lysine residue to transfer the acyl group to the thiol of CoA, producing an acyl-CoA. As a result, the lipoic arm becomes reduced. The E3 subunit (dihydrolipoamide dehydrogenase) then reoxidizes the lipoic arm, coupled to NADH production.

Regulation of α-ketoacid dehydrogenases is important for numerous physiological processes including metabolic switching during fasting and feeding, the response to hypoxia, and differentiation and has broad relevance to many diseases including cardiovascular conditions, cancer, diabetes, and inborn errors of metabolism^1,3^. The activity of α-ketoacid dehydrogenases is controlled through layers of transcriptional, post-translational, and metabolite-driven mechanisms. The best-known mechanisms include inhibitory phosphorylation of the E1 subunit of PDHC and BCKDC, which is controlled by specific kinases and phosphatases^4^. Post-translational modifications of the E2 and E3 subunits, including modification of the E2 subunit’s lipoic arms by glutathionylation, reactive oxygen species (ROS), and lipid peroxidation products, as well as cysteine oxidation and nitrosylation of the E3 subunit, have also been reported to modulate the activity of α-ketoacid dehydrogenase complexes^5–10^.

We recently found that PDHC and OGDC become profoundly inhibited in macrophages upon stimulation with the classical activation signals lipopolysaccharide (LPS) and interferon-γ (IFN-γ)^11^. This inhibition drives substantial TCA cycle remodeling, impacting the abundance of important immunomodulatory metabolites. We found that such PDHC and OGDC inhibition is largely due to loss of the catalytically active lipoic arm on their E2 subunits^11^. (Note, here we use ‘lipoic arm’ to refer to both the oxidized and reduced lipoic moiety, that is ‘lipoyl moiety’ and ‘dihydrolipoyl moiety’, respectively.) The molecular mechanism causing this loss was unclear.

Here we show that RNS, which macrophages produce in large amounts upon classical activation, are capable of substantially inhibiting PDHC and OGDC by causing a series of covalent S-modifications on their lipoic arm. CoA, the thiol-containing natural substrate for PDHC and OGDC’s E2 subunits, can deliver modifications to the lipoic arm, making this mechanism particularly efficient and specific. In activated macrophages, inhibiting RNS production significantly rescues the loss of PDHC and OGDC's functional lipoic arms and activities, and alters TCA cycle remodeling. This work reveals a novel mechanism regulating lipoic arm-dependent enzymes and provides a new mechanistic link between RNS and cellular metabolism that has broad implications for physiological and pathological conditions involving RNS accumulation.

## Results

### NO drives PDHC and OGDC inhibition by altering lipoic arm

**Fig. 1:**
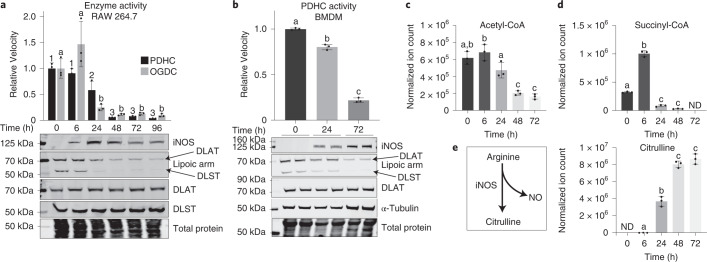
NO production temporally correlates with the loss of catalytically active lipoic arm and PDHC and OGDC activity. **a**, PDHC and OGDC activity, DLAT and DLST level and their lipoylation state, and iNOS level in RAW 264.7 cells stimulated with LPS + IFN-γ for the indicated time. **b**, PDHC activity, iNOS expression, protein lipoylation state and total DLAT level in murine BMDM stimulated with LPS + IFN-γ for the indicated time. **c**–**e**. The abundance of intracellular acetyl-CoA (**c**), succinyl-CoA (**d**) and citrulline (**e**) in BMDM stimulated with LPS + IFN-γ for the indicated time. ND, not detected. All bars and error bars represent mean ± s.d. (*n* = 3 distinct samples). Data were analyzed using one-way analysis of variance (ANOVA) followed by Tukey’s post hoc test. Bars not sharing a letter/number are significantly different (*P* < 0.05) from one another. Exact *P* values for each comparison are available in the source data. **a**,**b**, On lipoic arm western blots, specific bands corresponding to the lipoic arm of DLAT and DLST are labeled based on the expected molecular mass and the position of DLAT and DLST bands, as determined by total DLAT/DLST blots. The blots shown are representative of at least two independent experiments. Source data

We recently discovered that stimulation of macrophages by LPS + IFN-γ led to a profound inhibition of PDHC and OGDC over time^11^. This inhibition is in large part due to the loss of the functional lipoic arm on their E2 subunits, dihydrolipoamide acetyl transferase (DLAT) and dihydrolipoamide succinyl transferase (DLST), respectively, although total DLAT and DLST levels remain steady (Fig. 1a,b), and the expression of E1 and E3 subunits, as well as cell viability, do not decrease^11^.

**Extended Data Fig. 1:**
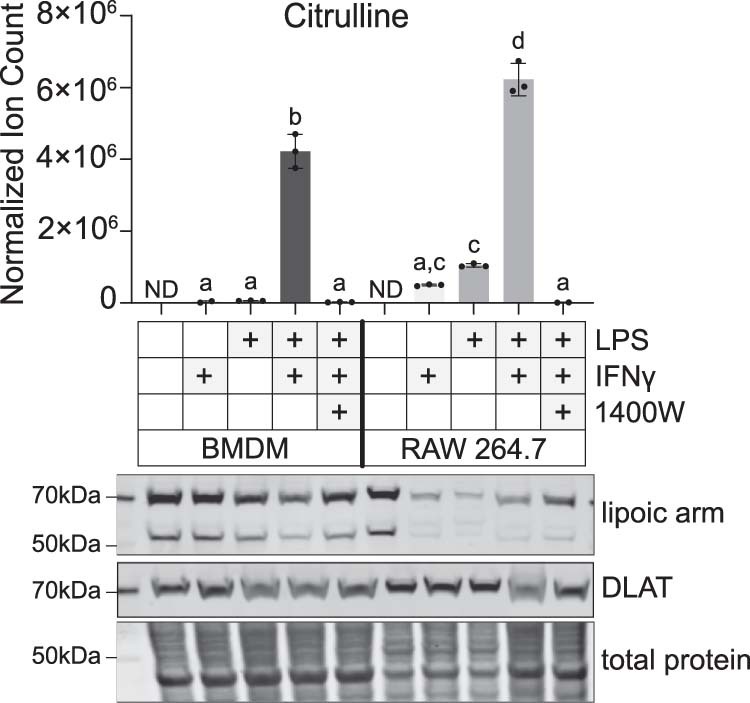
Loss of functional lipoic arm in conditions with high citrulline accumulation. Intracellular accumulation of the iNOS product citrulline, total DLAT level, and lipoic arm status in RAW 264.7 cells and murine bone marrow derived macrophages (BMDM) treated with various combinations of LPS, IFNγ and the iNOS inhibitor, 1400W. Bar graph and error bars represent mean ± SD, n=3 distinct samples (in each of the following two conditions, BMDM +IFNγ and RAW 264.7 + LPS + IFNγ + 1400W, citrulline was not detected in one of the 3 samples, so two dots are shown). ND indicates not detected. Data were analyzed using one-way ANOVA followed by Tukey’s post hoc test. Those bars not sharing a letter are significantly different (p<0.05) from one another. Exact *P*-values for each comparison are available in Source Data. Western blot data is representative of two independent experiments. Source data

Upon classical activation, macrophages quickly express inducible nitric oxide synthase (iNOS) to generate RNS for pathogen killing^12^. We noticed that the decrease in the functional lipoic arm and enzyme activity are temporally correlated with the increase in iNOS expression in two macrophage cell models, RAW 264.7 cells (Fig. 1a) and murine bone marrow-derived macrophages (BMDM) (Fig. 1b). Consistently, the intracellular levels of PDHC’s product acetyl-CoA and OGDC’s product succinyl-CoA also decreased over time (Fig. 1c,d), in temporal correlation with the accumulation of citrulline, the product of iNOS (Fig. 1e). Furthermore, across different stimulation conditions, we found that only when iNOS activity was considerable (indicated by noticeable citrulline accumulation), was there substantial loss of functional lipoic arms on PDHC and OGDC’s E2 subunit (Extended Data Fig. 1). Together, these results demonstrate a correlation between nitric oxide (NO) production and the loss of PDHC and OGDC’s functional lipoic arms and enzyme activities.

**Fig. 2:**
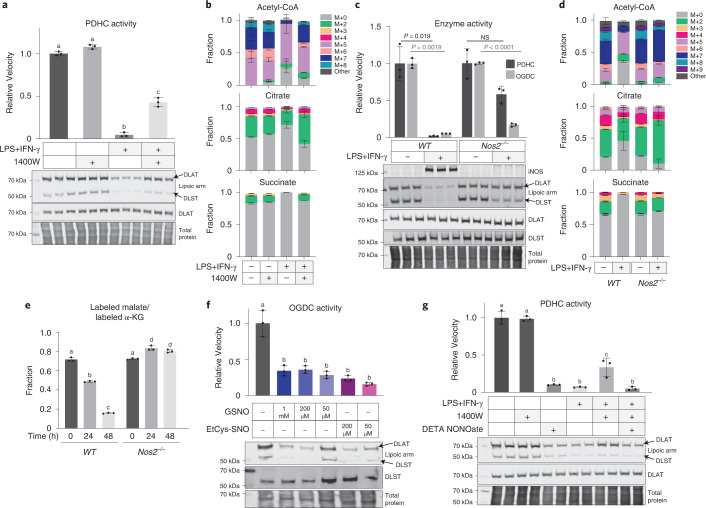
Loss of PDHC and OGDC activity and their functional lipoic arm depends on NO. **a**, Relative PDHC activity and lipoylation state in unstimulated or LPS + IFN-γ stimulated (48 h) RAW 264.7 cells with or without treatment with iNOS inhibitor 1400W (80 µM). Relative activity was normalized to the PDHC activity in unstimulated, untreated cells. **b**, Labeling patterns of acetyl-CoA, citrate and succinate after incubation with [U-^13^C]glucose tracer for 24 h in RAW 264.7 cells with or without LPS + IFN-γ stimulation and with or without 1400W for 48 h. M + i indicates the fraction of isotopologue with i-labeled carbons. **c**, Relative PDHC and OGDC activity and protein lipoylation state in unstimulated and LPS + IFN-γ stimulated (48 h) wild-type (*WT*) and *Nos2* knockout RAW 264.7 cells (*Nos2*^−*/*−^*)*. Relative activity was normalized to the respective unstimulated condition. *P* values were determined using the two-tailed Student’s *t*-test. NS, not significant (*P* > 0.05). **d**, Labeling patterns of acetyl-CoA, citrate and succinate after incubation with [U-^13^C]glucose tracer for 24 h in unstimulated or LPS + IFN-γ stimulated wild-type or *Nos2*^−*/*−^ RAW 264.7 cells. M + i indicates the fraction of isotopologue with i-labeled carbons. **e**, Ratio of the labeled malate fraction to the labeled α-KG fraction after incubation with [5-^13^C]glutamine tracer for 24 h in wild-type or *Nos2*^−*/*−^ RAW 264.7 cells stimulated with LPS + IFN-γ for the indicated length of time. **f**, OGDC activity and protein lipoylation state in the lysate of unstimulated RAW 264.7 cells with or without in vitro treatment of NO donor GSNO or EtCys-SNO at the indicated concentrations (3 h, room temperature). **g**, PDHC activity and protein lipoylation state in RAW 264.7 cells treated with the indicated combination of LPS + IFN-γ, iNOS inhibitor (80 µM 1400W) or NO donor (200 µM DETA NONOate) for 48 h. All bars and error bars represent mean ± s.d. (*n* = 3 distinct samples). **a**,**e**–**g**, Data were analyzed using one-way ANOVA followed by Tukey’s post hoc test. Those not sharing a letter are significantly different (*P* < 0.05) from one another. Exact *P* values for each comparison are available in the source data. **b**,**d**, Statistical comparison (ANOVA and Tukey’s post hoc test) between each labeled form is provided in the source data. Western blots shown in **a**, **c**, **f** and **g** are representative of at least two independent experiments. Source data

**Extended Data Fig. 2:**
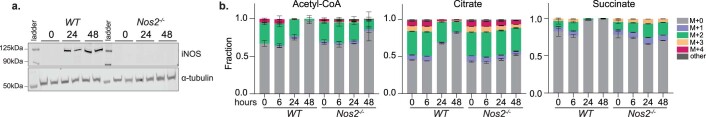
Remodeling of glucose metabolism through TCA cycle in LPS+IFNγ stimulated BMDM is largely NO dependent. **a**. iNOS level in wild-type and *Nos2*^−*/*−^ BMDMs stimulated with LPS+IFNγ for indicated time. α-tubulin levels are shown as a loading control. **b**. The labeling patterns of acetyl-CoA, citrate, and succinate after incubation with 1,2-^13^C-glucose tracer for 24h in wild-type or *Nos2*^−*/*−^ BMDM stimulated with LPS+IFN-γ for indicated time. All bars and error bars represent mean ± SD, (n=3 distinct samples). Statistical comparison (ANOVA followed by Tukey’s post-hoc test) between each labeled form is provided in Source Data. Source data

To test the causal relationship behind this correlation, we first treated RAW 264.7 cells with the iNOS inhibitor 1400W. Inhibition of iNOS significantly protected cells against the stimulation-induced loss in functional lipoic arms and PDHC activity (Fig. 2a). Consistently, iNOS inhibition also rescued the contribution of PDHC to acetyl-CoA production in stimulated macrophages, as reflected by the incorporation of [U-^13^C]glucose into acetyl-CoA, especially in the labeled forms that presumably involve labeling on the acetyl-moiety (for example, M + 7 labeled form) (Fig. 2b). This rescue of acetyl-CoA labeling was accompanied by a rescue of labeling in the downstream TCA cycle metabolites citrate and succinate. These data indicate that NO has a substantial impact on the remodeling of glucose oxidation through the TCA cycle in stimulated macrophages (Fig. 2b). We further tested the impact of NO using a genetic knockout of iNOS (*Nos2*^−*/*−^). Like 1400W treatment, iNOS knockout in RAW 264.7 cells significantly rescued the loss of PDHC and OGDC activity and their functional lipoic arms that occurs upon stimulation (Fig. 2c), and rescued the labeling incorporation from [U-^13^C]glucose into acetyl-CoA, citrate and succinate (Fig. 2d). Similar rescue of glucose labeling into these TCA cycle intermediates was also observed in stimulated *Nos2*^−*/*−^ BMDM cells (Extended Data Fig. 2). We additionally investigated the role of NO in regulating OGDC activity in cells using a [5-^13^C]glutamine tracer. [5-^13^C]Glutamine generates ^13^C-labeled α-KG, whose further conversion to malate through the TCA cycle is dependent on OGDC activity. In wild-type cells, as previously shown, the relative labeling incorporation into malate compared with α-KG decreases with LPS + IFN-γ stimulation, consistent with reduced OGDC activity^11^. This decrease, however, is prevented by iNOS knockout (Fig. 2e). These data collectively show that inhibition of PDHC and OGDC, loss of their functional lipoic arms, and the resulting impact on TCA cycle rewiring in activated macrophages are substantially dependent on NO production.

Next, using treatment with NO donors, we tested whether NO was sufficient to cause the loss of PDHC and OGDC activity and their functional lipoic arms. Treating cell lysates with *S*-nitrosoglutathione (GSNO) or *S*-nitroso-l-cysteine ethyl ester (EtCys-SNO) at concentrations as low as 50 µM caused significant inhibition of OGDC activity, as well as decreased levels of the functional lipoic arm relative to total DLST (Fig. 2f). Moreover, treating unstimulated macrophages with the NO donor diethylenetriamine NONOate (DETA NONOate) caused a reduction in functional lipoic arm and PDHC activity without significantly reducing total DLAT level or causing cellular toxicity (Fig. 2g and Supplementary Fig. 1), recapitulating what is observed in stimulated macrophages. Treatment with NO donor also reversed the 1400W-mediated protection of PDHC’s lipoic arm and activity in stimulated macrophages (Fig. 2g). Together, these results demonstrate that NO plays a key role in the loss of functional lipoic arms and the profound inhibition of PDHC and OGDC.

### RNS cause inhibitory S-modifications of the lipoic arm

We next investigated the molecular mechanism by which NO leads to loss of the functional lipoic arm. To evaluate the possibility that NO may inhibit lipoic arm installation or increase lipoic arm removal (both mechanisms would cause the E2 subunit to be unlipoylated), we measured the relative level of DLAT that is unlipoylated on its key lysine residue by LC–MS. HAP1 cells with a knockout of mitochondrial trans-2-enoyl-CoA reductase, an enzyme required for lipoic acid (LA) synthesis, were used as a positive control because the majority of DLAT is expected to be unlipoylated in these cells (confirmed by western blot in Supplementary Fig. 2a). Although LPS + IFN-γ stimulation can cause >95% activity loss of PDHC, the relative portion of DLAT that is unlipoylated (indicated by the measured level of unlipoylated peptide containing the key lysine residue normalized to the level of a reference DLAT peptide not containing the lipoylation site) was far below that observed in mitochondrial trans-2-enoyl-CoA reductase knockout cells, but similar to wild-type HAP1 (Supplementary Fig. 2b). Furthermore, although stimulation-induced PDHC inhibition can be largely rescued by iNOS inhibition, iNOS inhibition did not cause a considerable decrease in unlipoylated DLAT (Supplementary Fig. 2b). This suggests that a decrease in the total lipoic arm is unlikely to account for the near-complete, largely NO-dependent, inhibition of PDHC in stimulated macrophages.

It has been reported that the lipoic arms on PDHC and OGDC’s E2 subunits can be modified on their thiol groups by reactive compounds including ROS and lipid peroxidation products. These modifications prevent the lipoic arm from going through the catalytic cycle and thus inhibit enzyme activity^6,7,10^. In general, thiol groups are also susceptible to reaction with RNS. Therefore, we hypothesized that RNS derived from NO can inhibit α-ketoacid dehydrogenase complexes by modifying their lipoic arms.

**Fig. 3:**
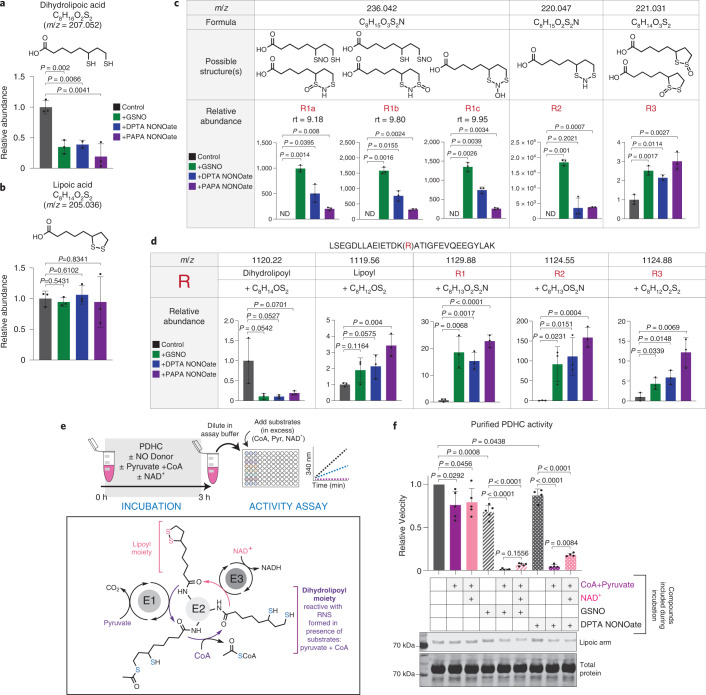
RNS cause S-modifications on lipoic arm and inactivate its catalytic activity. **a**,**b**, Abundance of DHLA (**a**) and LA (**b**) after incubation (3 h, room temperature) with or without NO donor, GSNO, DPTA NONOate or PAPA NONOate. **c**, Formulas, possible structures and relative abundances of major products after DHLA was incubated with the indicated NO donor (3 h, room temperature). Identified products with a unique formula are designated by R#. *m*/*z* is the exact mass of a singly charged negative ion. ND, not detected; rt, retention time. **d**, Depletion of substrate and appearance of products after dihydrolipoyl peptide LSEFDLLAEIETDK(dihydrolipoyl)ATIGFEVQEEGYLAK was incubated with the indicated NO donors in the presence of CoA (3 h, room temperature). *m*/*z* is the exact mass of the 3+ ion, R# corresponds to the modifications identified in **c**. In this LC condition the different isomers of R1 are not well separated by chromatography. Bars and error bars represent mean ± s.d. (*n* = 3 distinct samples). *P* values were determined using one-way ANOVA followed by Dunnett’s multiple comparison test. For species not detectable in controls, a one-sample two-sided Student’s *t*-test was used to test whether the mean was significantly different from 0. **e**, Schematic of experimental workflow and the catalytic mechanism of PDHC. After incubation with the indicated compounds, enzyme activity was measured after dilution into assay buffer containing all substrates at saturating levels. **f**, Activity and lipoylation state of purified PDHC following 3 h incubation (room temperature) with NO donors in the presence or absence of the indicated substrates. Independent experiments were combined and normalized to their own control (PDHC incubated in buffer). Bar graph with error bars represents mean ± s.d. *n* = 4 distinct samples for DPTA NONOate treatment, otherwise *n* = 5 distinct samples. For comparison between NO donor-treated samples, *P* values were determined by ANOVA followed by Tukey’s post hoc test. For comparison with the control (PDHC incubated in buffer), *P* values were determined using a one-sample *t*-test to determine whether the mean is significantly different from 1. The shown western blot is representative of two independent experiments. Source data

To evaluate the chemical reactivity of the lipoic arm to RNS, we first incubated free LA (C_8_H_14_O_2_S_2_) and its reduced form, dihydrolipoic acid (DHLA, C_8_H_16_O_2_S_2_), with NO donors, GSNO (which can release NO as well as react through *trans*-nitrosylation)^13,14^, dipropylenetriamine NONOate (DPTA NONOate) or propylamine NONOate (PAPA NONOate). We found that DHLA was significantly depleted after 3 h incubation with each NO donor, suggesting DHLA can nonenzymatically react with RNS. By contrast, oxidized LA, which does not contain free thiols, did not react with RNS (Fig. 3a,b). Untargeted LC–MS analysis revealed that the reaction between DHLA and RNS yielded a variety of products. The formulas of the most abundant products were identified based on their exact masses and isotopic distributions, and the likely structures were assigned based on previous knowledge of the chemical reactions between thiols and RNS^15–17^. These products included: (1) at least three distinct species, separated by their retention time, with the formula C_8_H_15_O_3_S_2_N, which correspond to *S*-nitrosylation of either thiol of DHLA, a hydroxyl-disulfenamide or a thiol-sulfinamide (R1); (2) C_8_H_15_O_2_S_2_N, corresponding to a disulfenamide product (R2); and (3) C_8_H_14_O_3_S_2_, corresponding to a thiosulfinate (R3) (Fig. 3c). These products are consistently observed in reactions with all three NO donors, although their abundances can vary depending on the specific RNS involved and RNS-to-thiol ratio^14–16^.

We next examined whether similar reactions can occur nonenzymatically when incubating NO donors with a synthesized dihydrolipoylated DLAT peptide [LSEGDLLAEIETDK(dihydrolipoyl)ATIGFEVQEEGYLAK]. We found that reaction with all three NO donors quickly depleted the peptide, generating a series of thiol modification products (Fig. 3d and Supplementary Fig. 3), corresponding to those observed in the free DHLA experiment (R1–R3).

We then tested whether PDHC could be inhibited by RNS in vitro, by incubating purified PDHC with NO donors. Because the results above showed that the reduced dihydrolipoic moiety, but not the oxidized lipoic moiety, can react with RNS, we also included different combinations of substrates during the incubation to manipulate the redox state of the lipoic arm. Based on the established catalytic mechanism of PDHC, inclusion of pyruvate and CoA can drive conversion of the lipoic moiety to its reduced form, and further addition of NAD^+^, the substrate of the E3 subunit, allows the lipoic arm to cycle between the reduced and oxidized forms (Fig. 3e). We found that when PDHC was incubated with NO donors in the presence of pyruvate and CoA, its activity was profoundly inhibited, accompanied by reduced functional lipoic arm levels. By contrast, incubation of PDHC with NO donors alone had a minimal impact on its activity (Fig. 3f). These results show that RNS can cause substantial inhibition of PDHC, and this inhibition requires the availability of thiols on the lipoic arm, indicating a specific mechanism involving lipoic thiol modification. Consistent with this model, when NAD^+^ was also added during the incubation period, and the lipoic arm was therefore in the reduced form for a shorter time, PDHC was also profoundly inhibited by RNS, but to a slightly lesser extent (Fig. 3f).

### *S*-Nitroso-CoA efficiently delivers modifications to the lipoic arm

**Fig. 4:**
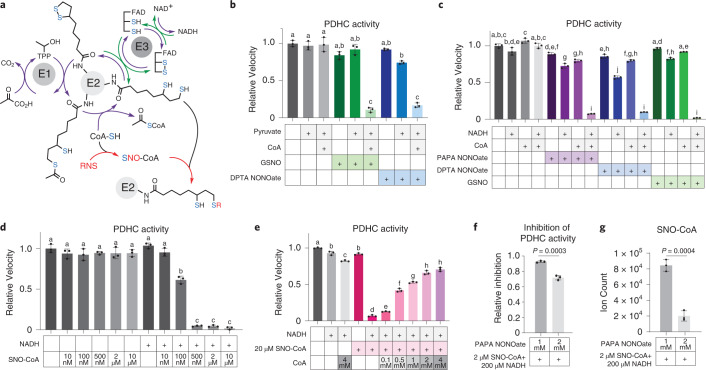
CoA delivers RNS modifications to the lipoic arm. **a**, Model schematic. CoA, the thiol-containing natural substrate of the E2 subunit, reacts with RNS and delivers modifications to the lipoic arm, blocking its catalytic cycle. Green arrows show that NADH can generate reduced lipoic arm via reversed E3 subunit activity. **b**, Activity of purified PDHC after 3 h incubation at room temperature with NO donors GSNO (400 µM) or DPTA NONOate (400 µM) in the presence or absence of CoA (400 µM) and/or pyruvate (400 µM). **c**, Activity of purified PDHC after 3 h incubation (room temperature) with NO donors GSNO (200 µM), DPTA NONOate (600 µM) or PAPA NONOate (600 µM) in the presence or absence of CoA (200 µM) and/or NADH (200 µM). **d**, Activity of purified PDHC after 1 h incubation with the indicated concentration of SNO-CoA in the presence or absence of NADH (200 µM). **e**, Activity of purified PDHC after 1 h incubation with 20 µM SNO-CoA in the presence or absence of NADH (200 µM) and varying amounts (as indicated) of CoA. **b**–**e**, Bars and error bars represent mean ± s.d., *n* = 3 different samples as indicated by individual dots. Data were analyzed using one-way ANOVA followed by Tukey’s post hoc test. Those not sharing a letter are significantly different (*P* < 0.05) from one another. Exact *P* values for each comparison are available in the source data. **f**,**g**, Relative inhibition of PDHC (**f**) after purified PDHC was incubated for 1 h (room temperature) with 2 µM SNO-CoA, 200 µM NADH and indicated dosages of additional PAPA NONOate. Relative inhibition is the loss of PDHC activity relative to PDHC control (incubated in buffer without substrate nor NO donor for 1 h). Actual abundance of SNO-CoA in each condition was measured by LC–MS and is shown in **g**. Bars and error bars represent mean ± s.d., *n* = 3 different samples as indicated by individual dots. Significance was determined by two-sided Student’s *t*-test. Source data

**Extended Data Fig. 3:**
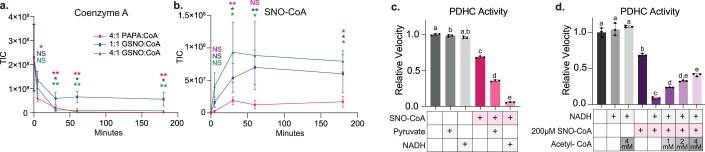
SNO-CoA delivers RNS modifications to the lipoic arm. **a**-**b**. The decrease in CoA level (a) and the appearance of SNO-CoA (b) over time after CoA was mixed with NO donor PAPA NONOate or GSNO at indicated ratio. Statistical difference between each time point comparing to time 0 was determined by Dunnett’s multiple comparisons tests. NS specifies not significant (p>0.05), * signifies p≤0.05, ** signifies p≤0.01. Color of significance indicator corresponds to respective condition with the same color. Exact *P*-values for each comparison are available in Source Data. Symbol and error bars represent mean ± SD, n=3 distinct samples. TIC, total ion count. **c**. The activity of purified PDHC after 3h incubation (RT) with ± 200µM SNO-CoA ± 200µM pyruvate ± 200µM NADH, as indicated on figure. **d**. The activity of purified PDHC after 1h incubation (RT) with 200µM SNO-CoA, 200µM NADH and acetyl-CoA at various doses as indicated. After incubation period and immediately prior to activity measurement, varying amount of acetyl-CoA was added to samples to even out the final acetyl-CoA concentration across all reactions. **c-d**. Bars and error bars represent mean ± SD, n=3 distinct samples. Data were analyzed using one-way ANOVA followed by Tukey’s post hoc test. Those bars not sharing a letter are significantly different from each other (p<0.05). Exact *P*-values for each comparison are available in Source Data. Source data

The specificity and stoichiometry of post-translational modifications have long been key topics in protein research^17–19^. Because most known ROS- and RNS-driven modifications are nonenzymatic, it is important to understand how some modifications can be sufficiently enriched at functionally relevant sites to impart important biological impacts. In this case, we found the NO-driven inhibition of PDHC can be almost complete both in vitro and in cells. We hypothesized that the key factor underlying such efficiency is CoA, the thiol-containing natural substrate for α-ketoacid dehydrogenases’ E2 subunits (Fig. 4a). It has been shown previously that low molecular mass thiol-containing metabolites, particularly glutathione and CoA, play an important role in mediating cysteine nitrosylation in cells by forming low molecular mass SNOs^17,19–21^. Indeed, we found that in the presence of various NO donors, *S*-Nitroso-CoA (SNO-CoA) is quickly and nonenzymatically formed from CoA (Extended Data Fig. 3a,b) (potential enzymatic formation may contribute further to SNO-CoA availability in cells). Based on the structural similarity between SNO-CoA and CoA, it is conceivable that SNO-CoA can efficiently bind to the E2 subunit and deliver RNS modifications to the lipoic arm in a targeted manner.

To test this hypothesis, we first examined whether CoA is required for RNS-driven PDHC inhibition. Although incubation of PDHC with pyruvate alone would also generate one free thiol on the lipoic arm, inclusion of pyruvate without CoA was not sufficient for the substantial inhibition of PDHC by NO donors (Fig. 4b). Alternatively, adding NADH to PDHC can generate reactive thiols on the lipoic arm through reversed activity of the E3 subunit (Fig. 4a, indicated by green arrow). When purified PDHC was incubated with NO donors in the presence of both NADH and CoA, we observed near-complete inhibition. By contrast, when either CoA or NADH was omitted during the incubation period the inhibition was much weaker (Fig. 4c). These results demonstrate that RNS-driven inhibition of PDHC depends on both the availability of reactive thiols on the lipoic arm and the presence of CoA. This requirement for CoA could, as hypothesized, reflect a role for SNO-CoA in delivering the modifications, or it is possible that CoA binding to the E2 subunit induces a conformational change allowing the lipoic arm to be more effectively modified.

To directly test the ability of SNO-CoA to inhibit PDHC, we incubated purified PDHC with SNO-CoA without additional CoA or other NO donors. SNO-CoA caused substantial inhibition in the presence of either pyruvate or NADH (Extended Data Fig. 3c). The inhibition of PDHC by SNO-CoA is dose-dependent, with 100 and 500 nM SNO-CoA causing ~50% and >95% inhibition after 1 h incubation (Fig. 4d), showing that SNO-CoA is a potent inhibitor of PDHC.

If SNO-CoA binding to the E2 subunit is key to the efficient delivery of inhibitory modifications, we would predict that competing with SNO-CoA binding can protect PDHC activity. Indeed, we found that addition of CoA reduced the inhibition of PDHC by SNO-CoA (20 µM) in a dose-dependent manner, with 0.5 and 4 mM CoA preventing ~50% and ~75% of the activity loss, respectively (Fig. 4e). Similarly, addition of acetyl-CoA, the natural product of DLAT which binds to the same site, also protected PDHC from inhibition by SNO-CoA in a dose-dependent manner (Extended Data Fig. 3d). These results not only illustrate that SNO-CoA binding is key to PDHC inhibition, but also highlight the potency of SNO-CoA-delivered inhibition. To preserve ~50% of PDHC activity over the 1 h incubation period, CoA or acetyl-CoA was required in large excess (>20-fold the SNO-CoA concentration). Such potency, along with the fact that unmodified lipoic arms can continue to react with SNO-CoA, allowing for accumulation of inhibitory modifications over time, suggests that a small amount of SNO-CoA in cells could be sufficient to cause significant inhibition.

Finally, we compared SNO-CoA against other NO donors in its ability to quantitatively control the inhibition of PDHC. If SNO-CoA is the primary compound delivering inhibitory modifications, we would expect the efficiency of PDHC inhibition to correlate with the SNO-CoA/CoA ratio. Alternatively, if the main mechanism of PDHC inhibition is direct reaction between RNS and the lipoic arm, we would expect the efficiency of PDHC inhibition to correlate more with the concentration of NO donor. To test this, we measured PDHC inhibition after incubation with a mixture of varying concentrations of GSNO and CoA, in the presence of pyruvate. LC–MS analysis revealed that when CoA is mixed with GSNO in solution, SNO-CoA is formed alongside other products (CoA-SG, CoA-CoA and so on). When the CoA concentration was relatively high compared with GSNO, increasing the GSNO concentration resulted in increasing SNO-CoA formation. However, when the CoA concentration was low, increasing GSNO caused SNO-CoA formation to first increase then decrease, with the yield of other products becoming more dominant when GSNO was present in large excess (Supplementary Fig. 4a–c). We found that the inhibition efficiency of PDHC showed a strong correlation with the SNO-CoA/CoA ratio, but not with the total level of GSNO across these conditions. When the CoA level is low, upon increasing the level of GSNO, PDHC inhibition first increased then substantially decreased; however, when the CoA level is high, PDHC inhibition remained strong across the dose curve (Supplementary Fig. 4d). Consistent with the observation that GSNO in a great excess of CoA reduces PDHC inhibition, we also found that although a low concentration of SNO-CoA in the presence of NADH can cause substantial inhibition of PDHC, adding a larger amount of the NO donor PAPA NONOate on top made the overall inhibition weaker (Fig. 4f). This seemingly counterintuitive decrease in inhibition correlates with a decreased level of SNO-CoA (Fig. 4g). Overall, these results show that the level of SNO-CoA, rather than other NO donors, has primary control of the inhibition, supporting a CoA-delivered modification mechanism.

### Reversibility and effects of RNS-driven PDHC/OGDC inhibition

**Fig. 5:**
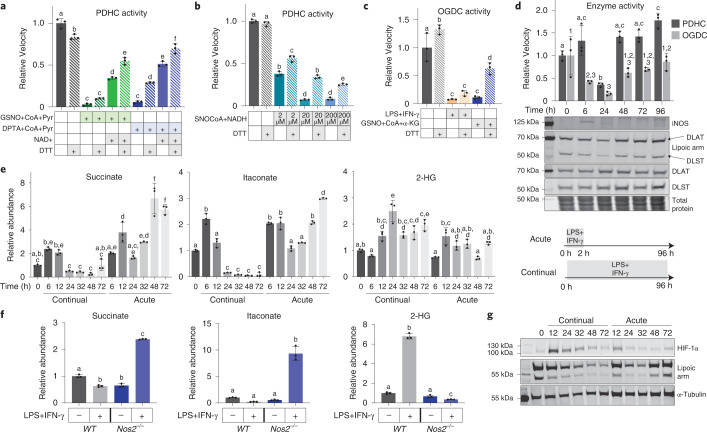
Recovery of RNS-mediated PDHC and OGDC inhibition in vitro and in cells. **a**, Activity of purified PDHC after incubation with indicated combinations of NO donors (GSNO or DPTA NONOate), CoA and pyruvate (3 mM each), and NAD^+^ (9 mM) followed by 30 min treatment with 10 mM DTT or buffer control. **b**, Activity of purified PDHC after incubation with or without SNO-CoA (at the indicated concentrations) and NADH (200 µM) followed by 30 min treatment of 10 mM DTT or buffer control. **c**, Changes in OGDC activity in cell lysate upon in vitro DTT treatment (20 mM). Lysates of unstimulated RAW 264.7 cells that had been incubated in vitro with the indicated combination of substrates and NO donor (GSNO, CoA and α-KG, 1 mM each), and lysate of LPS + IFN-γ stimulated (48 h) RAW 264.7 cells were treated with DTT or buffer control. Relative activity is normalized to OGDC activity in the lysate of unstimulated macrophages. **d**, PDHC and OGDC activity, total E2 subunit levels and their lipoylation status, and iNOS expression in RAW 264.7 cells over a time course following 2 h acute stimulation with LPS + IFN-γ. Blots are representative of two independent experiments. **e**, Changes in intracellular succinate, itaconate and 2-HG levels in RAW 264.7 cells over a time course either following 2 h acute stimulation with LPS + IFN-γ (acute) or with continual exposure to LPS + IFN-γ (continual). **f**, Intracellular succinate, itaconate and 2-HG levels in wild-type and *Nos2*^−*/*−^ RAW 264.7 cells with or without continual LPS + IFN-γ stimulation for 48 h. **a**–**f**, Bar graphs and error bars represent mean ± s.d., *n* = 3 distinct samples. Statistical analyses were done using one-way ANOVA followed by Tukey’s post hoc test. Those bars not sharing a letter/number are significantly different (*P* < 0.05) from one another. Exact *P* values for each comparison are available in the source data. **g**, Changes in HIF-1α level and protein lipoylation status in RAW 264.7 cells over a time course following 2 h acute stimulation with LPS + IFN-γ (acute) or continual exposure to LPS + IFN-γ (continual). α-Tubulin levels are shown as a loading control. The blot is representative of three independent experiments. Source data

Our data revealed that RNS can cause a diversity of thiol modifications on the lipoic arms (Fig. 3). Previous research has shown that the reversibility of different thiol modifications can vary. Modifications such as *S*-nitrosylation are generally reversible, regulatory and can be removed in cells by reducing agents such as glutathione or thioredoxin^22^. However, highly oxidized modifications are generally irreversible and thus can have long-lasting, even injurious, effects^23^. To begin to understand the reversibility of RNS-driven inhibition of α-ketoacid dehydrogenases, we investigated whether their activity could be recovered by in vitro treatment of a common reducing agent, DTT. We first tested this using purified PDHC that had been inhibited by incubation with either NO donors and substrates (Fig. 5a), or with SNO-CoA and NADH (Fig. 5b). DTT treatment generally resulted in the partial recovery of PDHC activity. However, the degree of recovery varied depending on the specific inhibition conditions, including the type of NO donor used, the substrates present during incubation (for example, whether NAD^+^ is present) (Fig. 5a) and the concentration of SNO-CoA (Fig. 5b). We next tested the extent to which treatment with DTT can recover OGDC activity in cell lysates (lysate from LPS + IFN-γ stimulated macrophage, and lysate that had been treated with NO donor in vitro). As with purified PDHC, DTT treatment partially recovered OGDC activity in cell lysates to various degrees (Fig. 5c). Together, these results show that the RNS-driven inhibition of α-ketoacid dehydrogenases is partially reversible, and the exact reversibility is condition-dependent. This likely reflects that overall inhibition of these enzymes is due to a diversity of inhibitory thiol modifications, whose composition varies with specific conditional parameters.

Next, using an acute stimulation model of macrophages we investigated whether and when RNS-driven PDHC and OGDC inhibition could be recovered in cells. In the experiments shown in Fig. 1, macrophages were cultured in the continual presence of LPS + IFN-γ (continual stimulation), which resulted in drastic and sustained induction of iNOS and NO production throughout the time course of the experiment. Alternatively, when macrophages were exposed to only LPS + IFN-γ for 2 h, then switched to fresh media without stimuli (acute stimulation), iNOS expression was only transiently induced, peaking at around 6 h and then returning to baseline throughout the rest of the time course (Fig. 5d), thus allowing for possible PDHC and OGDC recovery. Consistent with continual stimulation (Fig. 1), upon acute stimulation we also observed a decrease in PDHC and OGDC activity and their functional lipoic arms starting soon after iNOS induction and peaking at 24 h post-stimulation. However, at later time points, after iNOS levels had subsided, we observed the recovery of both PDHC and OGDC activity and their functional lipoic arms (Fig. 5d).

The kinetics of PDHC and OGDC inhibition and recovery can have important impacts on cellular metabolism and influence cell functions by altering the levels of signaling or regulatory metabolites. Several recent studies have highlighted that in macrophages, itaconate and succinate can orchestrate immune functions via mechanisms that include the stabilization of transcription factor hypoxia-inducible factor-1α (HIF-1α), modulation of nuclear factor-erythroid factor 2-related factor 2 (NRF2) and activating transcription factor 3 (ATF3) pathways, regulation of inflammasome activation, and modulation of the epigenetic landscape^24–28^. At early time points following LPS + IFN-γ activation, macrophages increase the expression of aconitate decarboxylase 1 (ACOD1 or IRG1), the enzyme that produces itaconate, causing itaconate to accumulate; itaconate-mediated inhibition of succinate dehydrogenase also causes succinate to accumulate^29–32^. We have previously shown that during continual stimulation following this initial accumulation, strong inhibition of PDHC and OGDC drives a decrease in itaconate and succinate levels by greatly restricting the influxes supporting their production^11^. Upon acute simulation in RAW 264.7 cells, we found that itaconate and succinate levels showed a similar trend, in which they initially accumulate and then decrease over the first 24–32 h (Fig. 5e), reflecting a similar rapid increase in ACOD1 expression and a gradual decrease in PDHC and OGDC activity during this period (Figs. 1a and 5d). Interestingly, in contrast to continual stimulation, under which these metabolites stay low for the rest of the time course (Fig. 1a), ~48 h after acute stimulation itaconate and succinate showed a strong second accumulation (Fig. 5e), consistent with the recovery of PDHC and OGDC activity at this time (Fig. 5d). Another important TCA cycle-derived regulatory metabolite is 2-hydroxy-glutarate (2-HG). In various other cell types, accumulation of 2-HG has been associated with OGDC inhibition or defects in the lipoic arm, and 2-HG accumulation can impact cell functions through stabilization of HIF and regulation of epigenetic remodeling^6,33–40^. We found that 2-HG levels accumulated across the continual stimulation time course, but were only slightly increased upon acute stimulation, peaking at 12 h (Figs. 1a and 5e).

To specifically test the role of NO in these metabolic changes, we measured the level of itaconate, succinate and 2-HG in *Nos2*^−*/*−^ RAW 264.7 cells continually stimulated for 48 h. In stark contrast to wild-type cells, where itaconate and succinate fell below baseline at this time point, in *Nos2*^−*/*−^ cells, itaconate and succinate remained highly elevated (Fig. 5f). This is consistent with our model that the decrease in itaconate and succinate is caused by NO-driven PDHC and OGDC inhibition, and is consistent with data showing that PDHC and OGDC activity and carbon incorporation from glucose and glutamine into the TCA cycle are rescued by iNOS knockout (Fig. 2c–e). iNOS knockout also prevented the accumulation of 2-HG upon stimulation (Fig. 5f). Together, these results demonstrate that the changes in these regulatory metabolites upon stimulation are impacted by NO production.

As mentioned above, one common mechanism by which itaconate, succinate and 2-HG impart their regulatory function, is via stabilization of HIF-1α by inhibiting its prolyl hydroxylation^6,11,35,38–41^. We have previously shown that during continual stimulation, the early transient increase in succinate and itaconate leads to a transient increase in HIF-1α stability and level^11^. This was followed by a decrease in HIF-1α levels as PDHC and OGDC become inhibited and itaconate and succinate levels fall. Similarly, here we found that upon acute stimulation of RAW 264.7 cells, HIF-1α levels first increased then decreased within the first 48 h. However, there was a second increase in HIF-1α levels at 72 h (Fig. 4g), following the second increase in itaconate and succinate. This suggests the fluctuation in succinate, itaconate and 2-HG, could influence the dynamic changes in HIF-1α upon both continual and acute stimulation. HIF-1α has been shown to promote proinflammatory phenotypes in macrophages^41–43^. This points to a possible mechanism by which the changing activity of PDHC and OGDC may contribute to tuning of the immune response.

## Discussion

In this work, we revealed that RNS can cause substantial inhibition of PDHC and OGDC, via a novel mechanism involving covalent thiol modification of their lipoic arms. We also demonstrate that CoA can mediate targeted delivery of RNS modifications onto the lipoic arm, making this mechanism particularly specific and efficient.

The experiments shown here provide strong biochemical evidence that RNS can cause inhibitory lipoic modifications and lay out several lines of supportive evidence that suggest an important role for this mechanism in metabolic rewiring during macrophage activation. However, a limitation of this work is the lack of direct measurement of these lipoic modifications in cells. Western blot-based measurement of the lipoic arm cannot distinguish whether the loss of detection is due to loss or modification of the lipoic arm, nor can it distinguish the specific types of modifications. The measurement is further complicated by the fact that multiple lipoylation sites on α-ketoacid dehydrogenase complexes cannot be separated on a gel, and that the antibody may still have some binding affinity toward some of the modified forms of the lipoic arm. We made substantial efforts to measure these RNS-driven lipoic modifications in cell lysates by LC–MS. However, the relative low abundance of DLAT and DLST, the chemical complexity of lipoic modifications, and stability issues with some of these modifications made measurement in cellular samples very technically challenging. Future development of a more sensitive method targeting the lipoic modifications is needed. With the help of such a method, one future direction is to quantitatively characterize how much each of the lipoic adducts is formed as a function of the RNS-to-thiol ratio, time, chemical environment and so on. In this study, we identified several types of covalent thiol modifications that have not previously been reported on the lipoic group. Although they should all block the lipoic arm’s catalytic activity, their reversibility can differ. Future work will also mechanistically address how the kinetics of PDHC and OGDC inhibition and recovery under specific conditions are affected by the quantitative distribution of these lipoic adducts, and to what extent the reduction of RNS-mediated modifications and the general turnover of PDHC and OGDC contribute to their recovery.

Here, we showed that PDHC and OGDC are profoundly inhibited in macrophages upon classical activation, and a considerable part of this inhibition is NO-dependent. Although the data suggest an important role of RNS-mediated lipoic modifications in driving this inhibition, several other potential contributing mechanisms are worth considering. First, it is possible that the lipoic arm may be removed from PDHC and OGDC’s E2 subunit. SIRT4 is the only enzyme known to be able to catalyze de-lipoylation^44^. We found that in macrophages after acute stimulation, *Sirt4* expression decreased substantially, then gradually recovered over time (Supplementary Fig. 5). This is opposite to the trend in PDHC inhibition and therefore unlikely to drive the loss of functional lipoic arm. It is also possible that synthesis or conjugation of the lipoic group may be reduced^45,46^. Such mechanisms would take effect only after the protein containing the already installed lipoic arm has been turned over. It has been shown that in phagocytes, PDHC has a half-life of >90 h^47,48^. Therefore, this mechanism is unlikely to be a driving factor in the strong inhibition of PDHC and OGDC occurring soon after iNOS induction, but might make some contribution at later time points upon continual stimulation. NO production may also lead to an increase in other related reactive species such as ROS, which can further contribute to the inhibition of PDHC and OGDC^5–8,10^. Finally, mechanisms targeting other subunits of PDHC or OGDC have been shown to also regulate their activity^1,5,9^. Our previous work showed that E1 subunit phosphorylation makes a minor contribution to PDHC inhibition in activated macrophages^11^, likely explaining the small portion of PDHC inhibition that cannot be fully recovered by iNOS knockout. Interestingly, a recent report identified that the E3 subunit of PDHC can be nitrosylated (the specific cysteine site was not identified), which inhibits its activity in activated macrophages^9^. We hypothesize that because the normal function of the E3 subunit is to reoxidize the lipoic arm using a Cys–Cys active site (Fig. 4a), it is likely that the E3 subunit can become modified on the catalytic cysteine via *trans*-nitrosylation with an NO-modified lipoic arm. This possible mechanistic connection between the E2 lipoic and E3 cysteine modifications merits further investigation, and could have synergizing effects that influence the efficiency, kinetics, and reversibility of RNS-mediated PDHC and OGDC inhibition.

In this study, we investigated the implications of NO-driven PDHC and OGDC inhibition in macrophages. However, the relevance of this mechanism may extend to a variety of cell types, including other cells that can produce NO, such as endothelial cells and neurons, and cells exposed to NO in the microenvironment^17,19,49^. This mechanism might also apply to other lipoic arm-dependent enzymes including another mitochondrial α-ketoacid dehydrogenase, BCKDC, and the glycine cleavage system, which controls branched-chain amino acid oxidation and mitochondrial one-carbon metabolism, respectively. Regulation of these enzymes has been shown to be critical for energy homeostasis, proliferation, differentiation and other cellular functions in various cell types, and has been implicated in numerous prevalent diseases^3,50^. The discoveries presented here therefore point to a broadly relevant novel mechanism for RNS to impact cellular physiology via inhibition of these key metabolic enzymes.

## Methods

### Cell culture

RAW 264.7 cells (ATCC) were cultured in RPMI 1640 with 25 mM HEPES, 1% penicillin/streptomycin and 10% FBS (dialyzed FBS was used for the metabolite extraction experiments) at 37 °C and 5% CO_2_. To stimulate the cells, 50 ng ml^−1^ LPS (*Escherichia coli* O111:B4, Sigma) and 10 ng ml^−1^ recombinant mouse IFN-γ (R&D Systems) were added to the media.

Murine BMDMs were isolated from wild-type C57BL/6J mice or B6.129P2-Nos2^*tm1Lau*^/J (*Nos2*^−*/*−^) mice (Jackson Lab), as indicated. Mice were bred and maintained according to protocols approved by the University of Wisconsin-Madison Institutional Animal Care and Use Committee. Mice were group housed on a standard 12 h light/dark cycle, the environmental conditions were maintained thermostatically at 18–23 °C with 40%–60% humidity, and mice were fed ad libitum and had continual access to drinking water. Bone marrow cells were harvested from femurs and tibia of 6–12-week-old mice (male and female). Cells were differentiated in RPMI 1640 containing 25 mM HEPES, 1% penicillin/streptomycin, 10% FBS and 20% L929 conditioned media as previously described^51^. From 3 d post isolation, media were changed every day until 7 d post isolation. Cells were then plated for experiments at 2.5 × 10^5^ cells ml^−1^ in RPMI 1640 containing 25 mM HEPES, 1% penicillin/streptomycin, 10% FBS (dialyzed used for metabolite extraction experiments) and 20 ng ml^−1^ recombinant mouse macrophage colony stimulating factor (R&D Systems). To stimulate BMDM, cells were incubated with 50 ng ml^−1^ LPS (*E. coli* O111:B4, Sigma) and 30 ng ml^−1^ recombinant mouse IFN-γ (R&D Systems).

To avoid nutrient depletion, media for both RAW 264.7 cells and BMDMs were refreshed every day until collection. For continual stimulation experiments, LPS and IFN-γ were maintained in the media throughout. For acute stimulation experiments, cells were cultured with LPS and IFN-γ for 2 h, after which stimuli were removed, cells were washed with Dulbecco’s PBS (dPBS), and cell culture was continued in fresh media without stimuli for the remaining time course.

In experiments in which cells were treated with iNOS inhibitor or NO donor, the following chemicals were added: the iNOS inhibitor 1400W (Sigma, 80 µM) and the NO donor DETA NONOate (Cayman Chemical, 200 µM). Potential cellular toxicity of DETA NONOate was determined by Cytotox Green staining using an IncuCyte live cell imager and analysis software (S3 2019, Sartorius)

For experiments involving stable isotopic tracing, cells were incubated with media containing [U-^13^C]glucose, [1,2-^13^C]glucose or [5-^13^C]glutamine (Cambridge Isotopes), which replaces the unlabeled glucose or glutamine in regular media at the same concentration. Cells were cultured with labeled media for 24 h before collection. Data from labeling experiments was adjusted for natural abundance of ^13^C.

### Generation of *Nos2*^−*/*−^ cells

To knockout *Nos2* in RAW 264.7 cells, 2 × 10^6^ cells were transfected via electroporation with 1 µM fluorescent *trans*-activating CRISPR RNA (tracrRNA, ITT, catalog no. ATTO550), 1 µM CRISPR RNA targeting mouse *Nos2* (crRNA, GTGACGGCAAACATGACTTC, IDT Design ID: Mm.Cas9.NOS2.1.AA) and 1 µM HiFi Cas9 enzyme (IDT, catalog no. 1081060) in 100 µl of Nucleofector Solution V plus supplement (Lonza, catalog no. VCA-1003), using the preprogrammed electroporation protocol D-032 on Nucleofactor II/2b. Cells were immediately plated on a 35-mm plate. Sixteen hours after transfection, cells positive for fluorescent tracrRNA were single-cell sorted by FACS (BD FACSAria III) onto a 96-well plate in 50% RAW 264.7 conditioned media. Single-cell colonies were expanded and subsequently screened for the lack of NO production following LPS + IFN-γ stimulation by measuring the appearance of nitrite in the media using a Greiss Reagent Kit (Promega, catalog no. G2930). Positive hits (colonies with high viability but with no nitrite production upon stimulation) were further validated by western blot for iNOS after 24 h stimulation with LPS + IFN-γ.

### Metabolite extraction

To extract intracellular metabolites, cells were washed three times with dPBS and metabolites were extracted with cold LC–MS grade 80:20 methanol/H_2_O (v/v). Samples were dried under nitrogen flow and subsequently dissolved in LC–MS grade water.

### Liquid chromatography mass spectrometry analysis of small molecules

For the analysis of cellular metabolites or chemical reactions between small molecules (the reaction between LA or DHLA with RNS, or the reaction between CoA and RNS), samples were analyzed using a Thermo Q-Exactive mass spectrometer coupled to a Vanquish Horizon UHPLC. Analytes were separated on a 100 × 2.1 mm, 1.7 µM Acquity UPLC BEH C18 Column (Waters), with a 0.2 ml min^−1^ flow rate and with a gradient of solvent A (97:3 H_2_O/methanol, 10 mM TBA, 9 mM acetate, pH 8.2) and solvent B (100% methanol). The gradient is: 0 min, 5% B; 2.5 min, 5% B; 17 min, 95% B; 21 min, 95% B; 21.5 min, 5% B. Data were collected in full-scan negative mode. Setting for the ion source were: 10 aux gas flow rate, 35 sheath gas flow rate, 2 sweep gas flow rate, 3.2 kV spray voltage, 320 °C capillary temperature and 300 °C heater temperature.

The metabolites reported here were identified based on exact *m*/*z* and retention times determined with chemical standards. Data were analyzed with MAVEN^52,53^. When ‘Normalized Ion Count’ is reported, total ion count values have been normalized to the protein content of that condition (as determined by BCA assay). When ‘Relative Abundance’ is reported, normalized ion count values have been normalized relative to unstimulated (0 h) controls.

### Analysis of the reaction between LA/DHLA and RNS

LA (Cayman Chemical) or DHLA (Sigma-Aldrich) was incubated with the NO donors, GSNO, DPTA NONOate and PAPA NONOate (Cayman Chemical) at room temperature in 20 mM sodium phosphate reaction buffer for 1 or 3 h, as indicated in figure legends. At the end of incubation, the reaction mixture was diluted in LC–MS grade methanol and immediately injected onto the LC–MS using the method outlined in the section ‘Liquid chromatography mass spectrometry analysis of small molecules’. To identify major products from the reaction between DHLA and RNS, LC–MS features were pulled from untargeted analysis of control (DHLA incubated without NO donor) and NO donor-treated samples. The features were systematically compared to find major peaks present at significantly higher levels in NO donor-treated samples, but not in control. These peaks were then clustered based on retention time and chromatographic profile to identify groups of unique chemical species and their parent peak (because each compound can give many LC–MS features that are isotopic peaks or adducts of the parent compound). The formulas were identified based on exact mass and the isotopic distribution, especially the natural S34 peak and C13 peaks that indicate their compositions.

### Analysis of reaction between lipoylated E2 peptide and NO donor

Dihydrolipoylated E2 peptide, LSEGDLLAEIETDK(dihyrdrolipoyl)ATIGFEVQEEGYLAK (>90% purity) was synthesized by GenScript. Because some of the dihyrdrolipoyl moiety would spontaneously oxidize to the lipoic moiety in solution, to reduce the disulfide bond before incubation with NO donors, the peptide (50 μM in 20 mM sodium phosphate buffer) was reduced using 25 mM tris(2-carboxyethyl)phosphine (70 °C for 20 min), and the tris(2-carboxyethyl)phosphine was then removed by stage-tip. Briefly, stage-tips were assembled by layering C18 Empore reversed-phase extraction disks (3 M) into the bottom of a 200-μl tip using a 16-guage needle and plunger. Tips were conditioned with 100 µl of 100% acetonitrile (ACN) (2,000*g*, 1 min) followed by 100 µl of 0.5% trifluoroacetic acid (TFA) (2,000*g*, 1 min). Reduced peptide sample was added to top of the stage-tip and then centrifuged at 500*g* for 2 min. The stage-tip was then washed four times with 150 µl of 0.5% TFA/5% ACN (2,000*g*, 2 min), and the sample was eluted using 100 µl of 70% ACN/0.5% TFA (2,000*g*, 3 min). The sample was then diluted in reaction buffer (20 mM sodium phosphate buffer containing 3 mM CoA). NO donors (GSNO, DPTA NONOate or PAPA NONOate) were added to the solution, incubated at room temperature for 1.5 h and subsequently analyzed by LC–MS.

Peptides were separated on a 100 × 2.1 mm, Accucore Vanquish UHPLC C18+ column (Thermo), with a flow rate of 0.25 ml min^−1^ and a gradient of solvent A (5:95 H_2_O/ACN) and solvent B (0.1% formic acid (FA)). The gradient is: 0 min, 90% B; 2 min, 90% B; 14 min, 1% B; 15 min, 1% B; 16 min, 90% B; 20 min, 90% B. Data were collected on a positive mode with full scan/ddMS2 (loop count: 3, isolation window: 2 *m*/*z*, fragmentation with a stepped NCE: 20, 25, 30) and analyzed using XCalibur v.4.0.

### SNO-CoA and EtCys-SNO synthesis

SNO-CoA and EtCys-SNO were freshly prepared following previously established protocols^21,54^, by reacting equal volumes of a 0.1 M CoA or cysteine ethyl ester in 1 M HCl with 0.1 M NaNO_2_ in water containing 100 µM EDTA and 100 µM DPTA.

### Activity assays of PDHC and OGDC in cell lysate

PDHC activity in cell lysate was analyzed using a pyruvate dehydrogenase enzyme activity microplate assay kit (Abcam), as per the manufacturer’s instructions. This kit measures PDHC activity by monitoring pyruvate-dependent NADH production. The NADH level was measured by absorbance of NADH-coupled dye (450 nm) using a BioTek Epoch2 plate reader. Data were analyzed using Gen5 TS v.2.09 software.

To evaluate OGDC activity, mitochondria were isolated and the α-KG-dependent succinyl-CoA production rate in mitochondria lysate was measured using LC–MS. To isolate mitochondria, cells were lysed in hypotonic lysis buffer (20 mM Tris buffer pH 7.4, 1 mM EDTA with phosphatase and protease inhibitors (Pierce protease and phosphatase inhibitor tablets)) and incubated on ice for 30 min. Lysate was then homogenized using a dounce homogenizer and separated via centrifugation at 700*g*, 4 °C, for 10 min, in a mannitol sucrose solution (440 mM mannitol/140 mM sucrose, 20 mM Tris, 1 mM EDTA, pH 7.4). The mitochondrial fraction was resuspended in hypotonic lysis buffer. Mitochondrial lysate (~30 µg protein) was diluted in 200 µl of assay buffer (1 mM MgCl_2_, 400 µM thiamine diphosphate, 50 mM HEPES, 10% glycerol, 0.05% BSA) and combined with substrates (α-KG, CoA, NAD^+^). Production of succinyl-CoA was then monitored by LC–MS by quenching the reaction with LC–MS grade methanol every 5–7 min over a 20–30 min time course. To measure succinyl-CoA, a similar method was used as indicated above in the section ‘Liquid chromatography mass spectrometry analysis of small molecules’; however, the gradient was accelerated for efficiency. The gradient is: 0 min, 5% B; 0.5 min, 5% B; 1 min, 30% B; 10 min, 75% B; 10.5 min, 95% B; 11 min, 95% B; 11.5 min, 5% B. The mean velocity of the reaction was blanked to a control containing no α-KG in the substrate solution and normalized relative to unstimulated or untreated control.

To investigate the ability of NO to inhibit PDHC or OGDC activity in isolated lysate, cell lysate (isolated using methods above) was incubated with NO donors at room temperature before activity measurement. The following NO donors were used: GSNO (Cayman Chemical), DPTA NONOate (Cayman Chemical) and EtCys-SNO (synthesized as above). The reversibility of RNS-mediated inhibition was tested by treating the lysate with an excess of DTT (20 mM) for 30 min (room temperature) after incubation with NO donors was complete.

### In vitro assays of purified PDHC

Purified porcine PDHC (Sigma) was used for in vitro assays of RNS-mediated inhibition of PDHC. Enzyme (20–100 mU ml^−1^) was incubated at room temperature with NO donors, GSNO, DPTA NONOate, PAPA NONOate (Cayman Chemicals) or SNO-CoA, along with the designated combinations of CoA, NAD^+^, pyruvate, NADH and acetyl-CoA (as indicated in figure legends) in 20 mM sodium phosphate buffer (pH 7.2). In experiments testing the reversibility of RNS-mediated PDHC inhibition, reactions were subsequently incubated with an excess of DTT (10 mM) or buffer control at room temperature for 30 min before measuring activity.

At the end of incubation, the reaction solution was further diluted 1:20 in assay solution containing thiamine pyrophosphate (100 µM), CoA (1–5 mM), pyruvate (1–5 mM) and NAD^+^ (2.5–50 mM). The concentrations of the substrates in assay conditions were chosen so that: (1) all substrates are at saturating levels so the maximal enzyme activity is measured; (2) the substrates added in assay buffers are in excess of any carried over substrates added during the incubation period (after the 1:20 dilution), so the variation in the final substrate concentrations in assay conditions across samples are negligible; and (3) the CoA level added in the assay condition is in excess of any leftover NO donor, so possible consumption of CoA by reaction with RNS would not significantly affect the assay. This was further verified by LC–MS analysis of the reaction mix. In experiments in which large amounts of acetyl-CoA were incubated with purified enzyme to test its protection against SNO-CoA-mediated inhibition, varying levels of acetyl-CoA were added back into the assay solutions after the incubation period, such that in the assay condition, the concentration of acetyl-CoA was even across samples to control for the possible effect of acetyl-CoA as a competitive inhibitor of PDHC.

PDHC activity was measured by the rate of NADH absorbance (340 nM) increase using a BioTek Epoch2 microplate reader. NADH production was read continuously, and mean velocity values were calculated from a linear portion of the curve (up to first 10–30 min depending on the experiment). Data were analyzed using Gen5 TS v.2.09 software.

### Gel electrophoresis and immunoblotting

Whole-cell lysate samples were prepared by lysing cells in RIPA buffer with phosphatase and protease inhibitors (Pierce protease and phosphatase inhibitor tablets) after washing three times with dPBS. The protein content of the lysate was quantified using a BCA assay kit (Thermo Fisher Scientific). Whole-cell lysates or purified PDHC reactions were separated on 4%–12% Bolt Gel (Thermo Fisher Scientific) after heat denaturation in sample buffer (Thermo Fisher Scientific). No reducing buffer was added to samples when probing for the lipoic arm to minimize the potential perturbation of thiol modifications on proteins. DTT was added to sample buffer when probing for other targets. Proteins were subsequently transferred to a 0.2-µM nitrocellulose membrane. Protein loading and transfer efficiency was determined using Revert total protein stain (Li-Cor). Membranes were blocked in 5% nonfat milk in TBS-T buffer and probed with the following primary antibodies at a 1:1,000 dilution: anti-HIF-1α (CST, catalog no. 14179S), anti-α-tubulin (Abcam, catalog no. ab7291), anti-pyruvate dehydrogenase E2 (DLAT; Abcam, catalog no. ab66511), anti-OGDH E2 (DLST; Abcam, catalog no. ab187699) anti-lipoic acid (Calbiochem, catalog no. 467695) and anti-iNOS (Thermo Fisher Scientific, catalog no. PA5-17100). Primary antibody incubation was followed by incubation with Li-Cor secondary antibodies (goat anti-rabbit 800CW, goat anti-mouse 680RD) at a 1:10,000 dilution. Membranes were imaged with a Li-Cor Odyssey ClX and quantified using Image Studio Lite software. On those gels blotted for lipoic arm, two major bands are observed at ~70 and ~50 kDa. The 70 kDa band matches the expected molecular mass of DLAT based on the sequence and band position of the total DLAT blot, although it may also overlap with lipoylation on E2E3BP, which is of a similar expected molecular mass. The 50 kDa band matches the expected molecular mass of DLST and the band position of total DLST blot, although this molecular mass can possibly overlap with lipoylation on BCKDC’s E2 subunit, dihydrolipoamide branched-chain transacylase.

### In-gel digestion and liquid chromatography mass spectrometry analysis

In-gel digestion was performed to target and separate the PDHC-E2 protein for MS analysis. Some 30 μg of Hap1 or RAW 264.7 protein lysate (quantified by BCA) was separated by SDS–PAGE. The gel was stained by AcquaStain Protein Gel Stain and protein bands corresponding to the molecular mass of PDHC-E2 were sliced and cut into pieces. Gel slices were placed in tubes for destaining with 30% ethanol, followed by dehydration with ACN and overnight digestion with 10 ng μl^−1^ trypsin/LysC protease in 100 mM Tris buffer. Digestion was stopped by adding 5% FA and the supernatant was transferred to new tubes. Further peptide extraction buffer of 1% FA in ACN/H_2_O (60:40, v/v) was added to the gel slices and aspirate to combine with previous supernatant after 10 min incubation. Extracted peptides were dried in SpeedVac and resuspended in 0.1% trifluoroacetic acid. Samples were desalted with C18 Omix tips before loading into the LC–MS system for analysis.

LC separation was performed using a Thermo Ultimate 3000 RSLCnano system. A 25 cm EASY-Spray PepMap C18 column (250 mm × 75 μm, 2 μm) was used at a flow rate of 300 nl min^−1^ with mobile phase A consisting of 0.1% FA in H_2_O, and mobile phase B consisting of 0.1% FA in ACN/H_2_O (80:20, v/v). An EASY-Spray source was used and temperature was set to 35 °C. Each sample was run with a 150 min gradient starting at 3% B for 4 min, increased to 25% B over the next 69 min, then further increased to 40% B over another 37 min, followed by 35 min at 99% B and decreased back to 3% B for equilibration for 5 min. An Acclaim PepMap C18 HPLC trap column (20 mm × 75 μm, 3 μm) was used for sample loading. MS detection was performed with Thermo Exploris 240 Orbitrap mass spectrometer in positive mode. The source voltage was set to 1,800 V, the ion transfer tube temperature was set to 275 °C and the RF lens was at 70%. Full MS spectra were acquired from *m*/*z* 350 to 1,400 at an Orbitrap resolution of 60,000, with the normalized AGC target of 300% (3E6) and auto maximum ion injection time. Data-dependent acquisition was performed at a cycle time of 3 s for precursors with a charge state of 2–6 at an isolated width of 2. The intensity threshold was 1 × 10^5^. Dynamic exclusion was 30 s with the exclusion of isotopes. Other settings for data-dependent acquisition include Orbitrap resolution of 15,000, HCD collision energy of 28% and auto maximum ion injection time.

Raw MS data files were analyzed by Proteome Discoverer v.2.5.0.400. A Sequest HT search engine was utilized for searching against either a human (Hap1 samples) or mouse (RAW 264.7 samples) protein database from UniProt. Dynamic modifications included in the search are: Met oxidation (+15.995 Da), Lys dihydrolipoylation (+190.049 Da), Lys lipoylation (+188.033 Da), N-terminal acetylation (+42.011 Da), N-terminal Met loss (−131.040 Da) and N-terminal Met loss + acetylation (−89.030 Da). The minimum peptide length was set to six amino acids. Precursor mass tolerance was 10 ppm and fragment mass tolerance was 0.02 Da. A Minora feature detector was used for peak detection in the processing step. Feature mapper and precursor ion quantifier nodes were used in the consensus step for protein and peptide quantification.

### Statistical analyses

Statistical analyses were conducted using GraphPad Prism 9.0 software, with each respective statistical test listed in the corresponding figure legend.

### Reporting summary

Further information on research design is available in the Nature Research Reporting Summary linked to this article.

## Online content

Any methods, additional references, Nature Research reporting summaries, source data, extended data, supplementary information, acknowledgements, peer review information; details of author contributions and competing interests; and statements of data and code availability are available at 10.1038/s41589-022-01153-w.

## Supplementary information

Supplementary Information Supplementary Figs. 1–5 and unprocessed blots for Supplementary Fig. 2.

Reporting Summary

## Data Availability

Source data are provided with this paper.

## Source data

Source Data Fig. 1 Statistical source data.
Source Data Fig. 1 Unprocessed western blots for Fig. 1.
Source Data Fig. 2 Statistical source data.
Source Data Fig. 2 Unprocessed western blots for Fig. 2.
Source Data Fig. 3 Statistical source data.
Source Data Fig. 3 Unprocessed western blots for Fig. 3.
Source Data Fig. 4 Statistical source data.
Source Data Fig. 5 Statistical source data.
Source Data Fig. 5 Unprocessed western blots for Fig. 5.
Source Data Extended Data Fig. 1 Statistical source data.
Source Data Extended Data Fig. 1 Unprocessed western blots for Extended Data Fig. 1.
Source Data Extended Data Fig. 2 Statistical source data for Extended Data Fig. 2b.
Source Data Extended Data Fig. 2 Unprocessed western blots for Extended Data Fig. 2a.
Source Data Extended Data Fig. 3 Statistical source data for Extended Data Fig. 3.

## Code availability

No custom code was used in this study.
