# Supplementary material for: Nitric oxide-driven modifications of lipoic arm inhibit α-ketoacid dehydrogenases
Source: Nat Chem Biol. 2022 Oct 20;19(3):265–74. doi: 10.1038/s41589-022-01153-w (PMC9974485; doi:10.1038/s41589-022-01153-w)
Supplement: Supplementary file 1 — Supplementary Figs. 1–5 and unprocessed blots for Supplementary Fig. 2. [file 41589_2022_1153_MOESM1_ESM.pdf]

nature chemical biology

Article https://doi.org/10.1038/s41589-022-01153-w

Nitric oxide-driven modifications of lipoic arm inhibit α-ketoacid dehydrogenases

In the format provided by the authors and unedited

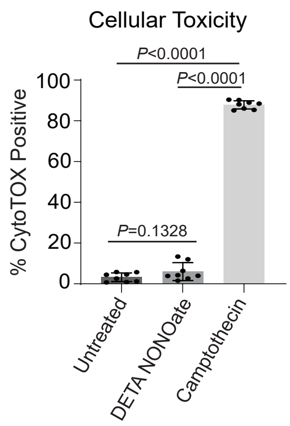

Supplementary Figure 1.
Cellular toxicity of 200μM DETA-NONOate treatment, as measured by the percentage of cells positive for CytoTOX green staining, in RAW 264.7 cells with or without DETA-NONOate treatment for 48h, corresponding to the condition in Figure 2g. Cells treated with camptothecin are shown as a positive control. Bar graph and error bars represent mean ± SD, n=8 distinct samples. Statistical analysis was performed by two-sided Student's t-test with *P*-value indicated on figure.

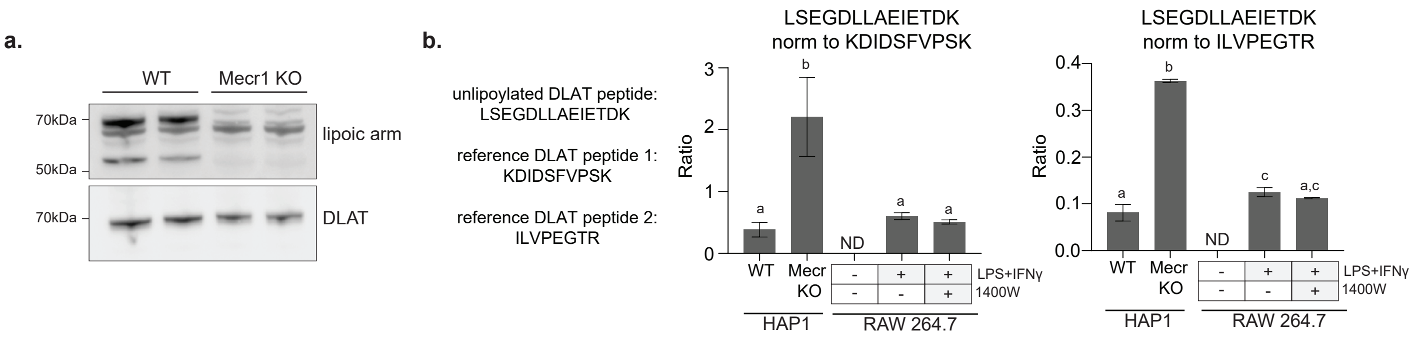

Supplementary Figure 2.

a. Total level and lipoic arm status of the E2 subunit of PDHC (DLAT) in wildtype and MECR knockout HAP1 cells. MECR is an enzyme required for the synthesis of lipoic acid. Western has been performed twice with similar results.

b. Ratio of unlipoylated DLAT peptide LSEGDLLAEIETDK to two indicated reference DLAT peptides in WT and MECR knockout HAP1 cells and unstimulated or LPS+IFNγ stimulated RAW 264.7 cells with or without treatment with iNOS inhibitor 1400W. Bars with error bars represent mean ± SD. For RAW 264.7 cells, n=3 distinct samples in each condition, however, in the condition stimulated with LPS+IFNγ and treated with 1400W, the targeted peptide was only detected in 2 out of the 3 samples. For HAP1 controls, n=2 distinct samples. ND indicates not detected. Data were analyzed using one-way ANOVA followed by Tukey's post hoc test. Those bars not sharing a letter are significantly different from each other (P<0.05).

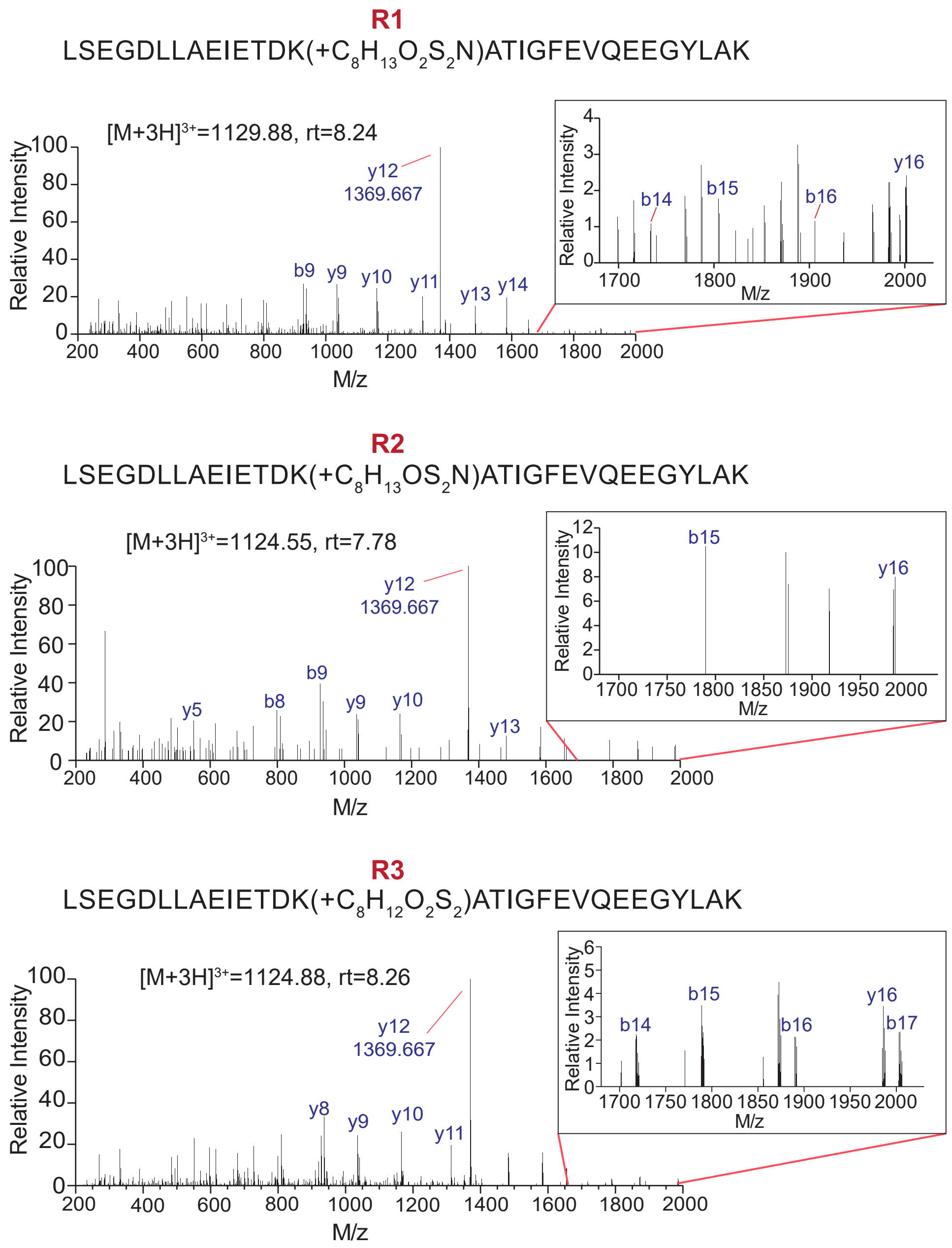

Supplementary Figure 3.

MS/MS spectra of major identified products of the dihydrolipoyl peptide LSEFDLLAEIETDK(dihydrolipoyl) ATIGFEVQEEGYLAK incubated with NO donor confirming their identity. R# corresponds to the identified lipoic modifications identified in Figure 3. rt= retention time, $[M+3H]^{3+}$ indicates exact mass of 3+ ion.

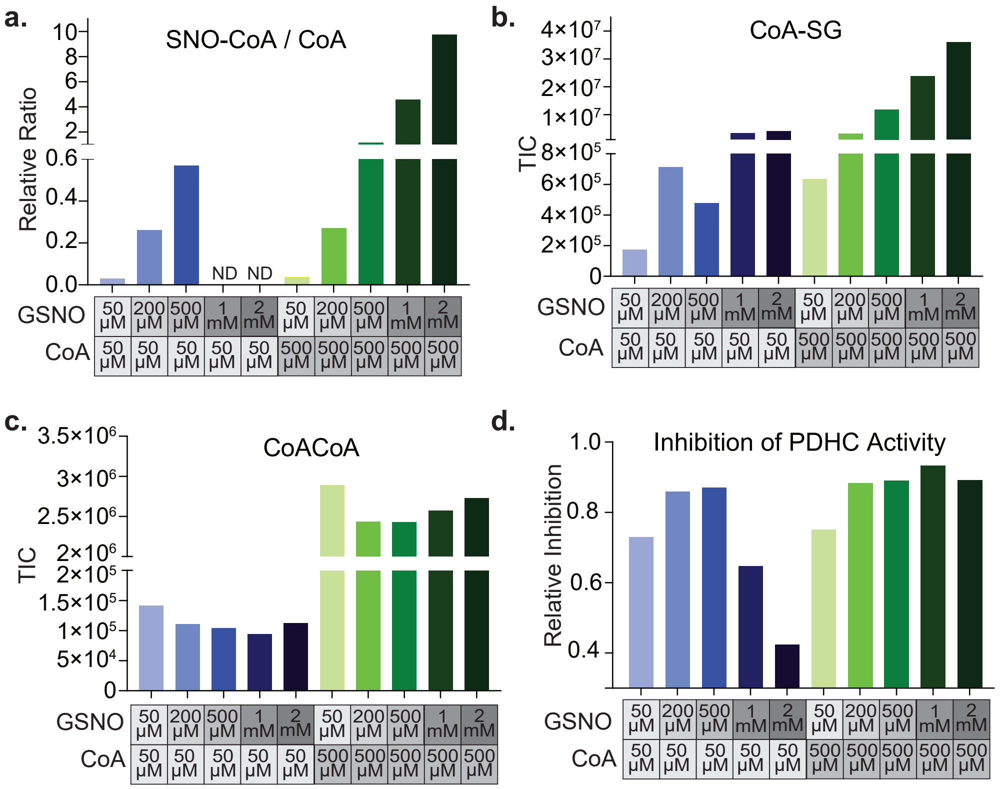

Supplementary Figure 4.
a-c. The relative ratio between SNO-CoA and CoA (a), the formation of CoA-glutathione (CoA-SG) (b) and the formation of CoA-CoA (c) when indicated doses of GSNO and CoA are mixed in solution. TIC = total ion count.
d. Relative inhibition of PDHC when purified PDHC was incubated for 3h (RT) with indicated dosages of GSNO and CoA in presence of 500µM pyruvate. Relative inhibition is the loss of PDHC activity relative to PDHC control (incubated in buffer without substrate nor NO donor).
a-d. Each bar represents data from n=1.

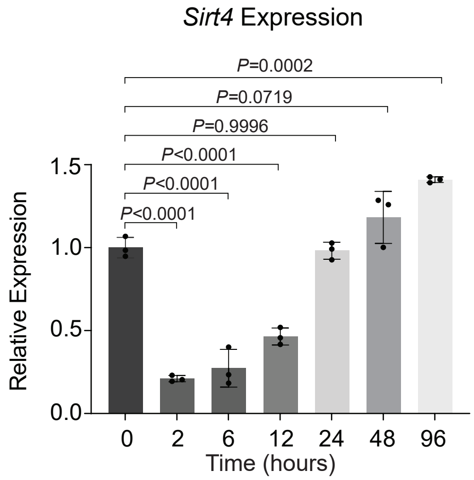

Supplementary Figure 5.

Relative expression of *Sirt4* mRNA in BMDMs at indicated time points after a 2h acute stimulation with LPS+IFNγ. Bars represent mean ± SD, n=3 different samples. Statistical significance comparing the difference between each time point against unstimulated (0h) control was determined by one-way ANOVA followed by Dunnett's multiple comparisons test.

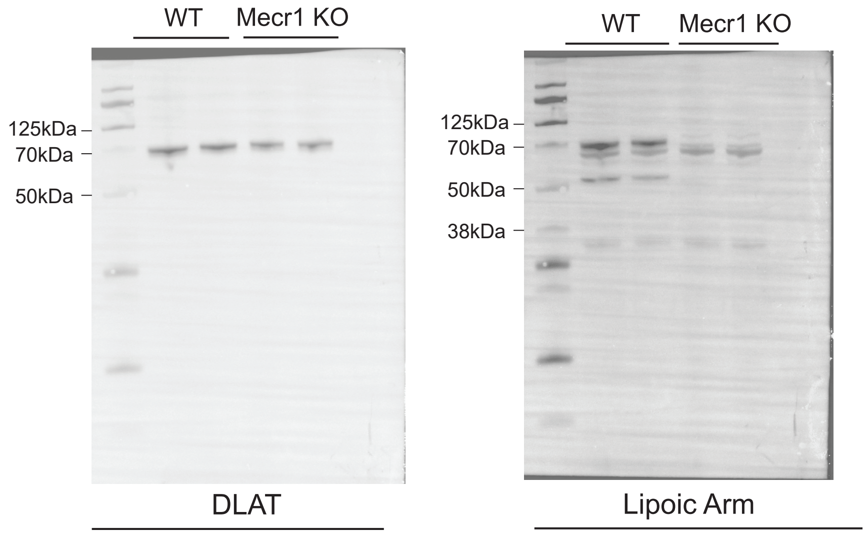

Uncropped Blots from Supplementary Figure 2
