# Supplementary material for: Nitric oxide-driven modifications of lipoic arm inhibit α-ketoacid dehydrogenases
Source: Nat Chem Biol. 2022 Oct 20;19(3):265–74. doi: 10.1038/s41589-022-01153-w (PMC9974485; doi:10.1038/s41589-022-01153-w)
Supplement: Supplementary file 2 — Reporting Summary [file 41589_2022_1153_MOESM2_ESM.pdf]

nature research

Corresponding author(s): Jing Fan

Last updated by author(s): Aug 17, 2022

# Reporting Summary

Nature Research wishes to improve the reproducibility of the work that we publish. This form provides structure for consistency and transparency in reporting. For further information on Nature Research policies, see our Editorial Policies and the Editorial Policy Checklist.

## Statistics

For all statistical analyses, confirm that the following items are present in the figure legend, table legend, main text, or Methods section.

| n/a | Confirmed | |
|---|---|---|
| ☐ | ☒ | The exact sample size ($n$) for each experimental group/condition, given as a discrete number and unit of measurement |
| ☐ | ☒ | A statement on whether measurements were taken from distinct samples or whether the same sample was measured repeatedly |
| ☐ | ☒ | The statistical test(s) used AND whether they are one- or two-sided<br>*Only common tests should be described solely by name; describe more complex techniques in the Methods section.* |
| ☒ | ☐ | A description of all covariates tested |
| ☐ | ☒ | A description of any assumptions or corrections, such as tests of normality and adjustment for multiple comparisons |
| ☐ | ☒ | A full description of the statistical parameters including central tendency (e.g. means) or other basic estimates (e.g. regression coefficient) AND variation (e.g. standard deviation) or associated estimates of uncertainty (e.g. confidence intervals) |
| ☐ | ☒ | For null hypothesis testing, the test statistic (e.g. $F$, $t$, $r$) with confidence intervals, effect sizes, degrees of freedom and $P$ value noted<br>*Give P values as exact values whenever suitable.* |
| ☒ | ☐ | For Bayesian analysis, information on the choice of priors and Markov chain Monte Carlo settings |
| ☒ | ☐ | For hierarchical and complex designs, identification of the appropriate level for tests and full reporting of outcomes |
| ☒ | ☐ | Estimates of effect sizes (e.g. Cohen's $d$, Pearson's $r$), indicating how they were calculated |

*Our web collection on statistics for biologists contains articles on many of the points above.*

## Software and code

Policy information about availability of computer code

| | |
|---|---|
| Data collection | XCalibur 4.0 software for LCMS data; Gen5 TS 2.09 software for Biotek microplate reader data; ImageStudio Lite Ver 5.2 (Licor) for western blot imaging; IncuCyte S3 2019, Sartorius for capturing live cell images |
| Data analysis | Graph Pad Prism 9.0 were used to perform statistical analyses; Maven version 6.2 (build 682) and XCalibur 4.0 were used to analyze LCMS data; ImageStudio Lite Ver 5.2 was used for western blot analysis; IncuCyte S3 2019, Sartorius for analyzing live cell images |

For manuscripts utilizing custom algorithms or software that are central to the research but not yet described in published literature, software must be made available to editors and reviewers. We strongly encourage code deposition in a community repository (e.g. GitHub). See the Nature Research guidelines for submitting code & software for further information.

## Data

Policy information about availability of data

All manuscripts must include a data availability statement. This statement should provide the following information, where applicable:

- Accession codes, unique identifiers, or web links for publicly available datasets
- A list of figures that have associated raw data
- A description of any restrictions on data availability

Source data files are provided along with the manuscript.

# Field-specific reporting

Please select the one below that is the best fit for your research. If you are not sure, read the appropriate sections before making your selection.

☒ Life sciences ☐ Behavioural & social sciences ☐ Ecological, evolutionary & environmental sciences

For a reference copy of the document with all sections, see nature.com/documents/nr-reporting-summary-flat.pdf

# Life sciences study design

All studies must disclose on these points even when the disclosure is negative.

| | |
|---|---|
| Sample size | No sample size calculation was performed prior to experiments; sample sizes were chosen based on typical standard in the field. |
| Data exclusions | No data was excluded from successfully executed experiments with the exception of Figure 3a and 3c (R3) where a replicate of the DPTA treated DHLA was excluded as an outlier using Grubb's test (P=0.1), details shown in Source data |
| Replication | All experiments have been successfully repeated at least twice |
| Randomization | Samples were randomly allocated to each treatment group |
| Blinding | Experiments were not conducted with blinding. Blinding was not possible as frequently one person was responsible for carrying out each experiment from beginning to data analysis. |

# Reporting for specific materials, systems and methods

We require information from authors about some types of materials, experimental systems and methods used in many studies. Here, indicate whether each material, system or method listed is relevant to your study. If you are not sure if a list item applies to your research, read the appropriate section before selecting a response.

## Materials & experimental systems

| n/a | Involved in the study | |
|---|---|---|
| ☐ | ☒ | Antibodies |
| ☐ | ☒ | Eukaryotic cell lines |
| ☒ | ☐ | Palaeontology and archaeology |
| ☐ | ☒ | Animals and other organisms |
| ☒ | ☐ | Human research participants |
| ☒ | ☐ | Clinical data |
| ☒ | ☐ | Dual use research of concern |

## Methods

| n/a | Involved in the study | |
|---|---|---|
| ☒ | ☐ | ChIP-seq |
| ☒ | ☐ | Flow cytometry |
| ☒ | ☐ | MRI-based neuroimaging |

## Antibodies

| | |
|---|---|
| Antibodies used | anti-HIF-1α (clone D2U3T, CST 14179), anti-α-tubulin (Abcam ab7291), anti-PDHC E2 (DLAT, Abcam ab66511), anti-OGDC E2 (DLST, Abcam, ab187699) anti-lipoic acid (Calbiochem, 467695), anti-iNOS (ThermoFisher PA5-17100), goat anti-rabbit 800CW (Licor, 926-32211 ) goat anti-mouse 680RD (Licor, 926-68070) --- all were used at a 1:1000 dilution |
| Validation | all had previously been validated by providers. HIF-1α: https://www.cellsignal.com/products/primary-antibodies/hif-1a-d2u3t-rabbitmab/14179; PDH E2: https://www.abcam.com/pyruvate-dehydrogenase-e2-antibody-ab66511.html lipoic acid: http://www.emdmillipore.com/US/en/product/Anti-Lipoic-Acid-Rabbit-pAb,EMD_BIO-437695#anchor_REF; iNOS: https://www.thermofisher.com/antibody/product/iNOS-Antibody-Polyclonal/PA5-17106; α-tubulin: https://www.abcam.com/alpha-tubulinantibody-dm1a-loading-control-ab7291.html; OGDH E2: https://www.abcam.com/dlst-antibody-ab187699.html |

## Eukaryotic cell lines

Policy information about cell lines

| | |
|---|---|
| Cell line source(s) | RAW 264.7 (ATCC® TIB-71™) |
| Authentication | RAW 264.7, which is a murine cell line, was not authenticated |
| Mycoplasma contamination | RAW 264.7 cell line regularly tested negative for mycoplasma |
| Commonly misidentified lines (See ICLAC register) | no commonly misidentified cell lines were used |

## Animals and other organisms

Policy information about studies involving animals; ARRIVE guidelines recommended for reporting animal research

| | |
|---|---|
| Laboratory animals | For isolation of BMDM the following animals were used:<br>Species: Mus musculus<br>Strains: C57BL/6J and B6.129P2-Nos2tm1Lau/J (homozygous)<br>Age: 6-12 weeks of age<br>Sex: male and females<br>Mice were group housed on a standard 12h light/dark cycle, the environmental conditions were maintained thermostatically between 18-23°C with 40-60% humidity and mice were fed ad libitum and had continual access to drinking water. |
| Wild animals | study did not involve wild animals |
| Field-collected samples | study did not involve field-collected samples |
| Ethics oversight | Mice were bred and maintained according to protocols approved by the University of Wisconsin-Madison Institutional Animal Care and Use Committee |

Note that full information on the approval of the study protocol must also be provided in the manuscript.
