# Supplementary figures and images for: Nitric oxide-driven modifications of lipoic arm inhibit α-ketoacid dehydrogenases
Source: Nat Chem Biol. 2022 Oct 20;19(3):265–74. doi: 10.1038/s41589-022-01153-w (PMC9974485; doi:10.1038/s41589-022-01153-w)

**Figure 1a**

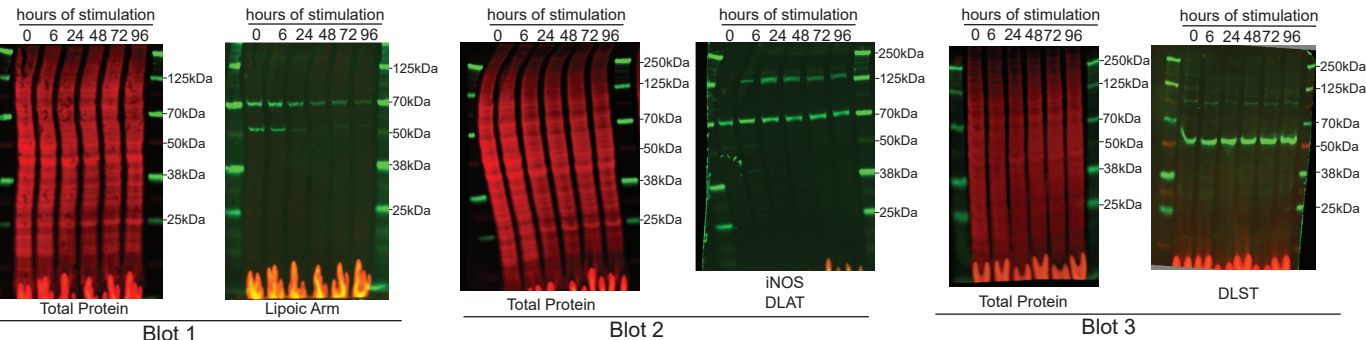

**Figure 1b**

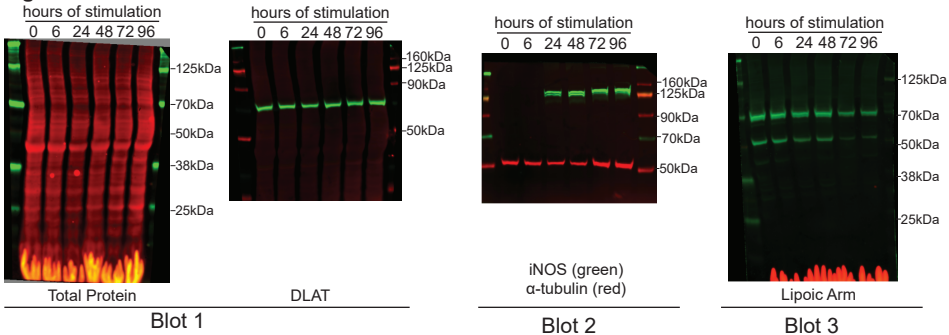

Supplement: Source Data Fig. 1 — Unprocessed western blots for Fig. 1. [file 41589_2022_1153_MOESM4_ESM.pdf]

Figure 2a

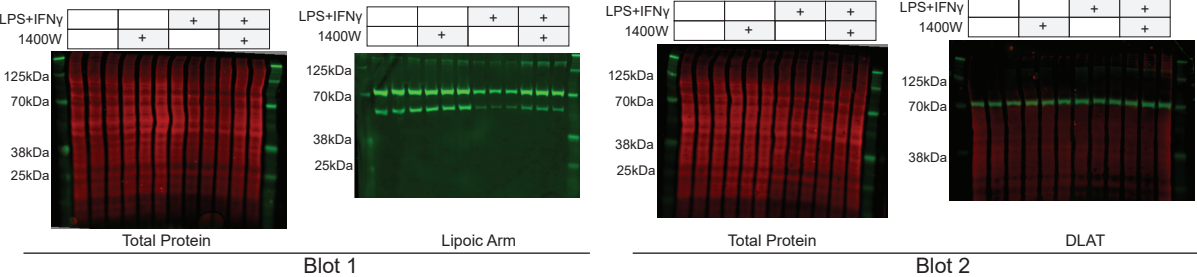

Figure 2c

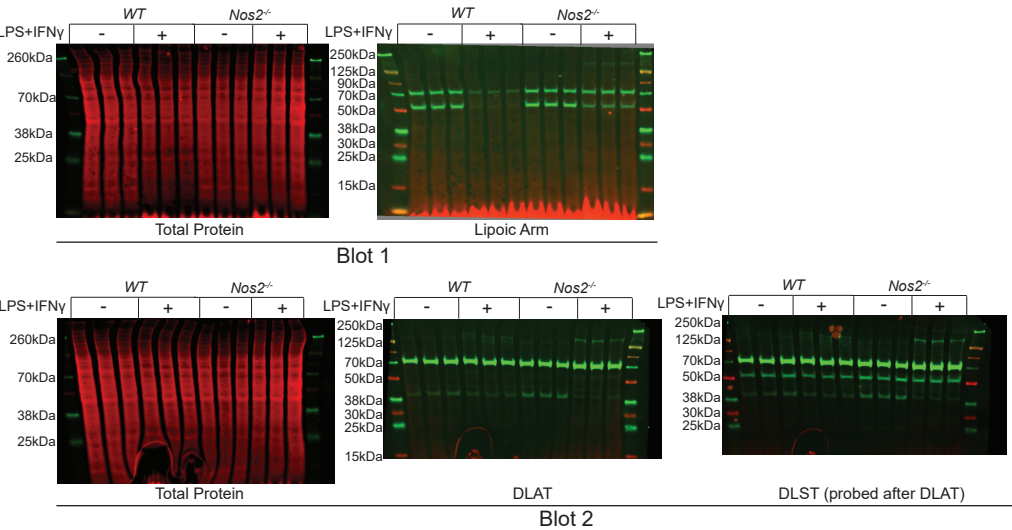

Figure 2f

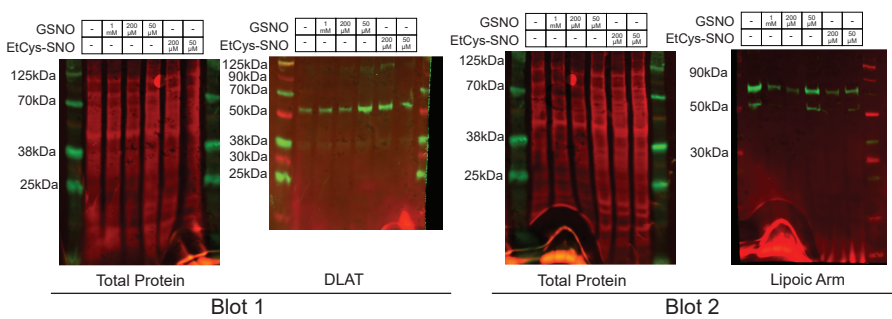

Figure 2g

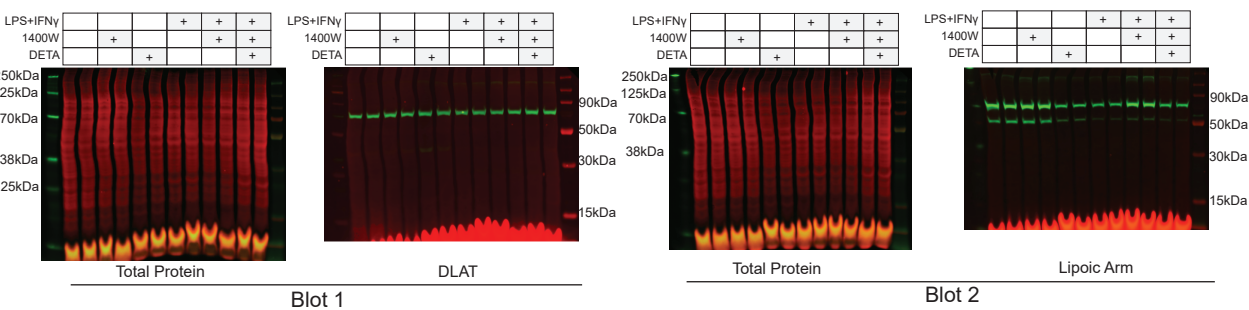

Supplement: Source Data Fig. 2 — Unprocessed western blots for Fig. 2. [file 41589_2022_1153_MOESM6_ESM.pdf]

Figure 3f

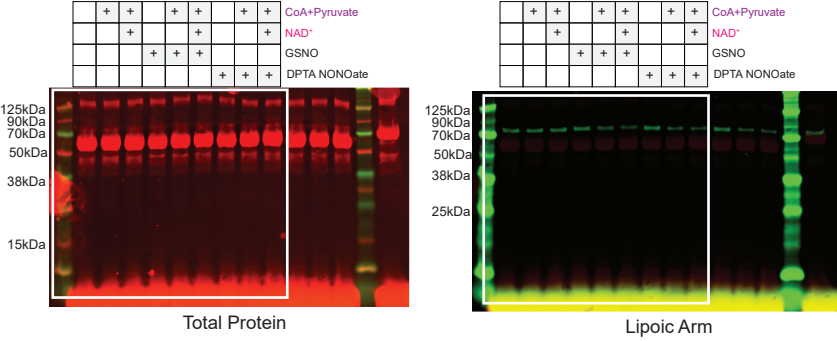

Supplement: Source Data Fig. 3 — Unprocessed western blots for Fig. 3. [file 41589_2022_1153_MOESM8_ESM.pdf]

Figure 5d

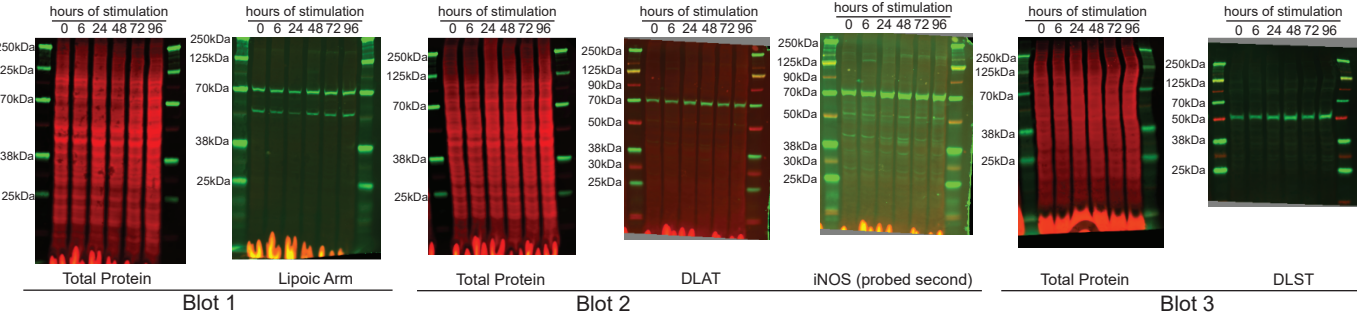

Figure 5g

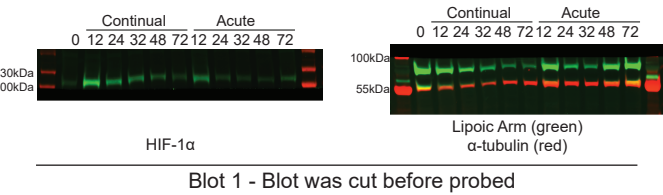

Supplement: Source Data Fig. 5 — Unprocessed western blots for Fig. 5. [file 41589_2022_1153_MOESM11_ESM.pdf]

Extended Data Figure 1

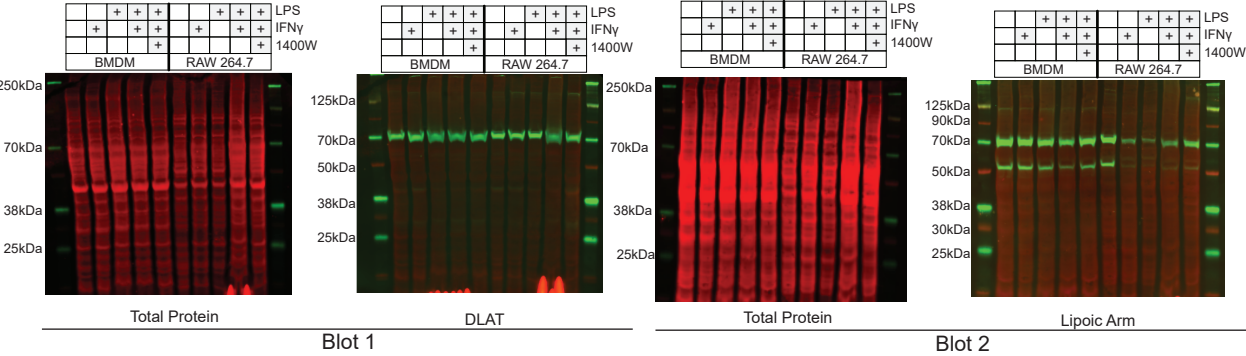

Supplement: Source Data Extended Data Fig. 1 — Unprocessed western blots for Extended Data Fig. 1. [file 41589_2022_1153_MOESM13_ESM.pdf]

Extended Data Figure 2

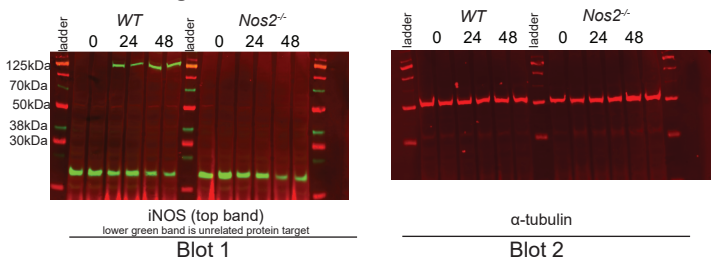

Supplement: Source Data Extended Data Fig. 2 — Unprocessed western blots for Extended Data Fig. 2a. [file 41589_2022_1153_MOESM15_ESM.pdf]
